# The Schmiedeberg Medal of the German Society for Experimental and Clinical Pharmacology and Toxicology: a biographical and bibliometric analysis of the 47 recipients from 1956 to 2024

**DOI:** 10.1007/s00210-025-04260-2

**Published:** 2025-06-07

**Authors:** Jessica Marie Steinert, Roland Seifert

**Affiliations:** Institute of Pharmacology, Hannover Medical School, Carl-Neuberg-Str. 1, 30625 Hannover, Germany

**Keywords:** Schmiedeberg Medal, Scientific award, Prize winner analysis, Bibliometric analysis

## Abstract

The Schmiedeberg Medal, awarded by the German Society for Experimental and Clinical Pharmacology and Toxicology (DGPT), is the highest honour for excellent pharmacologists and has been awarded to 47 recipients until 2024. The first medal was awarded to Wolfgang Heubner in 1956, the most recent of our analysis to Franz Hofmann in June 2024. This is the first profound analysis of the prize winners: Most of the prize winners (63.83%) were born in Germany, and more than half (53.19%) came from academic families. Notably, only two women (4.26%), Edith Bülbring and Marthe Vogt, have received the award, although the presence of women in medicine and among members of the DGPT is on the rise. Almost 90% of the award winners studied medicine, and 29.79% spent part of their studies in Berlin. The award winners enjoyed exceptional quality of life and consistently surpassed the average life expectancy, with an average age of 86.26 years. On average, their careers spanned 50.65 years, with the Schmiedeberg Medal being awarded 20.74 years after the peak of their careers. Over time, the number of publications and productivity, co-authors, and senior authorships increased, while first authorships decreased. This shift reflects the growing importance of collaborative scientific work as opposed to solo endeavours. The overall publication productivity also increased during the observation period, with the *Naunyn–Schmiedeberg’s Archives of Pharmacology* being the most prominent journal for these publications, although its influence decreased over time. The predominant language of publication was English, accounting for 72.28% of the articles, a trend that has increasingly replaced German since the 1970s. The most common research focus was the cholinergic and adrenergic systems. Thus, Schmiedeberg Medal recipients are an outstandingly productive group of scientists enjoying a long scientific career and a long high-quality life. In addition, Schmiedeberg Medal recipients encountered numerous societal and scientific challenges which they mastered excellently. The gender gap for this award is dramatic, reflecting the very low number of eligible female pharmacologists for this award.

**Supplementary Information:**

The online version contains supplementary material available at 10.1007/s00210-025-04260-2.

## Introduction

In addition to the gratification of a scientific career through publications and rankings, there is also the possibility of receiving a scientific prize for one’s achievements. The highest award for pharmacologists in the German-speaking world is the Schmiedeberg Medal, awarded by the DGPT for exceptional achievements in pharmacological research (Flockerzi and Biel 2025; Lutz et al. 2025; Martin et al. 2025). Despite its prominence, there has been no profound research examining the profile of the prize winners and their journey to scientific excellence. The goal of our study was to explore the factors and circumstances that lead to the awarding of this distinguished prize.

The origin of the Schmiedeberg Medal is well documented in the records of the board meetings of the German Pharmacological Society (precursor of the DGPT): After the tragic death of German pharmacologist and pioneer of modern anaesthesiology Hellmut Weese in January 1954, the German Pharmacological Society wanted to establish a foundation in the name of their respected member Weese. Its intended goal was to promote and support German pharmacologists and improve scientific research. However, after some consideration and planning, the board of directors realized that the financial state of the society did not allow to establish the “Stiftung Weese”. Instead, Werner Schulemann suggested to implement a scientific award instead (Protocol of the board meeting of the German Pharmacological Society, Mainz, 04.12.1954: ArchMHH Dep. 13 Nr. 2). The award should not include any monetary contribution, so the society would retain their finances and still be honouring their high achieving members. Schulemann also suggested that the award be named after Oswald Schmiedeberg, former student of Rudolf Buchheim, with whom he laid the foundation for modern pharmacology (Philippu and Seifert 2023). In the board meeting of July 2, 1955 “…they (the board) propose(d) to create a Schmiedeberg Prize which can be awarded to deserving members of the society for special achievements by the general meeting. Such certificates of honour should only be issued from time to time at irregular intervals and under special circumstances” (Protocol of the board meeting of the German Pharmacological Society, Bonn, 02.07.1955: ArchMHH Dep. 13 Nr. 2). About one year later, on August 4, 1956, the first Schmiedeberg medal was awarded to Wolfgang Heubner, former student of Schmiedeberg and professor emeritus of pharmacology at the University of Berlin (Philippu 2017). Gustav Kuschinsky, the society’s chairman at the time, presented him the medal at the International Congress of Physiologists in Brussels, to which Heubner replied: “What am I supposed to do with this? I already have one of those!”. Later, Heubner apologized for this rude behaviour and claimed to have had hypoglycaemia after administering insulin shortly beforehand, which allegedly caused his confusion (Letter from G. Kuschinsky to H.P. Wolf, 14.10.1987: ArchMHH Dep. 13 Nr. 13). Despite these initial difficulties, the award has been successfully presented to 47 pharmacologists until 2024, 4 of whom have also received a Nobel Prize (Otto Loewi, Henry H. Dale, Ulf von Euler, Julius Axelrod) (Table 1, Table S1).

**Table 1:** Key data of the 47 Schmiedeberg Medal prize winners from 1956 to 2024: by year of award

	Last name	First name(s)	Year of award	Gender	Date of birth	Date of death	Country of birth	Highest education of at least one parent	Course of study	Cities of study	Topic of research (Seifert 2019)
1	Heubner	Wolfgang	1956	Male	18.06.1877	26.02.1957	Germany	Science graduate	Medicine	Göttingen, Berlin, Marburg, Straßbourg	Introduction and Pharmacodynamics
2	Loewi	Otto	1957	Male	03.06.1873	25.12.1961	Germany	Skilled occupation	Medicine	Straßbourg, Munich	Cholinergic and Adrenergic System
3	Pick	Ernst Peter	1957	Male	18.05.1872	15.01.1960	Czech Republic	Skilled occupation	Medicine	Prague	Immunopharmacology
4	Dale	Henry Hallett	1962	Male	09.06.1875	23.07.1968	England	Skilled occupation	Science	London	Cholinergic and Adrenergic System
5	Heymans	Corneille	1962	Male	28.03.1892	18.07.1968	Belgium	Science graduate	Medicine	Gent	Drugs for Treatment of Hypertension
6	Liljestrand	Göran	1962	Male	16.04.1886	16.01.1968	Sweden	Unknown	Medicine	Stockholm	Drugs for Treatment of Respiratory Tract Diseases
7	Schmidt	Carl Frederic	1962	Male	29.07.1893	04.04.1988	USA	Skilled occupation	Medicine	Pennsylvania	Cholinergic and Adrenergic System
8	Holtz	Peter	1964	Male	06.02.1902	09.11.1970	Germany	Skilled occupation	Medicine	Heidelberg, Würzburg, Freiburg, Munich, Bonn	Cholinergic and Adrenergic System
9	Krayer	Otto	1964	Male	22.10.1899	18.03.1982	Germany	Common labour	Medicine	Freiburg, Munich, Berlin	Cholinergic and Adrenergic System
10	Schaumann	Otto	1965	Male	14.04.1891	24.01.1977	Austria	Unknown	Medicine	Vienna	Cholinergic and Adrenergic System
11	Verney	Ernest Basil	1967	Male	22.08.1894	19.08.1967	England	Common labour	Science	Cambridge	Cholinergic and Adrenergic System
12	Burn	Joshua Harold	1967	Male	06.03.1892	13.07.1981	England	Skilled occupation	Science	Cambridge	Cholinergic and Adrenergic System
13	von Euler	Ulf	1968	Male	07.02.1905	09.03.1983	Sweden	Science graduate	Medicine	Stockholm	Cholinergic and Adrenergic System
14	Feldberg	Wilhelm Siegmund	1968	Male	19.11.1900	23.10.1993	Germany	Skilled occupation	Medicine	Hamburg, Berlin, Munich	Cholinergic and Adrenergic System
15	Brodie	Bernard B	1969	Male	07.08.1907	28.02.1989	England	Skilled occupation	Chemistry	New York	Introduction and Pharmacodynamics
16	Schulemann	Werner	1969	Male	04.05.1888	20.06.1975	Germany	Skilled occupation	Medicine, Chemistry	Freiburg, Breslau	Drugs for Treatment of Fungal Infections
17	Blaschko	Hermann	1972	Male	04.01.1900	18.04.1993	Germany	Science graduate	Medicine	Freiburg, Berlin	Cholinergic and Adrenergic System
18	Buelbring	Edith	1974	Female	27.12.1903	05.07.1990	Germany	Graduate	Medicine	Bonn, Munich, Freiburg	Cholinergic and Adrenergic System
19	Dost	Friedrich Hartmut	1974	Male	11.07.1910	02.11.1985	Germany	Unknown	Medicine	Rostock, Freiburg, Leipzig, Innsbruck	Pharmacokinetics
20	Vogt	Marthe Louise	1974	Female	08.09.1903	09.09.2003	Germany	Science graduate	Medicine, Chemistry	Berlin	Cholinergic and Adrenergic System
21	Kosterlitz	Hans Walter	1976	Male	27.04.1903	26.10.1996	Germany	Science graduate	Medicine	Berlin, Heidelberg, Freiburg	Pain Pharmacology
22	Wilbrandt	Walther	1977	Male	11.01.1907	25.07.1979	Germany	Skilled occupation	Medicine	Tübingen, Kiel, Freiburg, Vienna, Berlin	Introduction and Pharmacodynamics
23	Schild	Heinz Otto	1977	Male	18.05.1906	15.06.1984	Croatia	Graduate	Medicine	Munich, Berlin	Introduction and Pharmacodynamics
24	Axelrod	Julius	1978	Male	30.05.1912	29.12.2004	USA	Common labour	Science	Washington	Cholinergic and Adrenergic System
25	Ariëns	Everhardus	1980	Male	29.01.1918	03.03.2002	Netherlands	Unknown	Chemistry, Medicine	Utrecht	Cholinergic and Adrenergic System
26	Herken	Hans	1981	Male	30.06.1912	21.03.2003	Germany	Unknown	Medicine	Düsseldorf	Introduction and Pharmacodynamics
27	Kuschinsky	Gustav	1982	Male	10.01.1904	17.11.1992	Germany	Skilled occupation	Medicine	Tübingen, Marburg, Berlin	Cholinergic and Adrenergic System
28	Remmer	Herbert	1985	Male	06.03.1919	23.06.2003	Germany	Skilled occupation	Medicine	Berlin, Jena	Pharmacokinetics
29	Fleckenstein	Albrecht	1987	Male	03.03.1917	04.04.1992	Germany	Graduate	Medicine	Würzburg, Vienna	Cholinergic and Adrenergic System
30	Reuter	Harald	1987	Male	25.03.1934	23.02.2022	Germany	Science graduate	Medicine	Freiburg, Innsbruck	Cholinergic and Adrenergic System
31	Hornykiewicz	Oleh	1994	Male	17.11.1926	26.05.2020	Ukraine	Graduate	Medicine	Vienna	Dopaminergic System
32	Brock	Norbert	1995	Male	26.05.1912	25.06.2008	Germany	Graduate	Medicine	Münster, Düsseldorf	Drugs for Treatment of Malignant Tumor Diseases
33	Lembeck	Fred	1995	Male	04.07.1922	22.10.2014	Austria	Graduate	Medicine	Vienna, Graz	Pain Pharmacology
34	Trendelenburg	Ullrich	1998	Male	31.12.1922	21.11.2006	Germany	Science graduate	Medicine	Göttingen, Uppsala	Cholinergic and Adrenergic System
35	Markwardt	Fritz	2000	Male	03.12.1924	10.09.2011	Germany	Graduate	Pharmacy, Medicine	Greifswald	Drugs for Treatment of Thromboembolic Diseases
36	Mutschler	Ernst	2002	Male	24.05.1931	-	Germany	Science graduate	Pharmacy, Medicine	Munich, Tübingen	Cholinergic and Adrenergic System
37	Vater	Wulf	2002	Male	27.11.1917	22.09.2007	Germany	Science graduate	Medicine	Cologne, Berlin	Drugs for Treatment of Hypertension
38	Muschaweck	Roman	2002	Male	15.12.1918	02.05.2007	Germany	Unknown	Medicine	Würzburg	Pharmacology of the Kidney
39	Eichelbaum	Michel	2008	Male	19.05.1941	-	Germany	Skilled occupation	Medicine	Heidelberg, Gießen	Pharmacokinetics
40	Muscholl	Erich	2010	Male	03.07.1926	17.01.2019	Germany	Science graduate	Medicine	Mainz	Cholinergic and Adrenergic System
41	Bock	Karl Walter	2012	Male	08.09.1935	-	Germany	Graduate	Pharmacy, Medicine	Freiburg, Berlin	Pharmacokinetics
42	Schroer	Karsten	2012	Male	03.05.1942	-	Germany	Science graduate	Medicine	Halle-Wittenberg	Drugs for Treatment of Thromboembolic Diseases
43	Göthert	Manfred	2014	Male	12.12.1939	28.06.2019	Germany	Science graduate	Medicine	Hamburg, Freiburg, Innsbruck, Vienna, Göttingen	Cholinergic and Adrenergic System
44	Schultz	Günter	2016	Male	23.01.1936	14.08.2021	Germany	Science graduate	Medicine	Berlin	Introduction and Pharmacodynamics
45	Philippu	Athineos	2022	Male	22.09.1931	-	Greece	Graduate	Medicine	Athens	Cholinergic and Adrenergic System
46	Starke	Klaus	2023	Male	01.11.1937	26.01.2024	Germany	Science graduate	Pharmacy, Medicine	Freiburg, Heidelberg	Cholinergic and Adrenergic System
47	Hofmann	Franz	2024	Male	21.05.1942	-	Austria	Science graduate	Medicine	Heidelberg, Munich, Berlin	Introduction and Pharmacodynamics

**Fig. 1:**
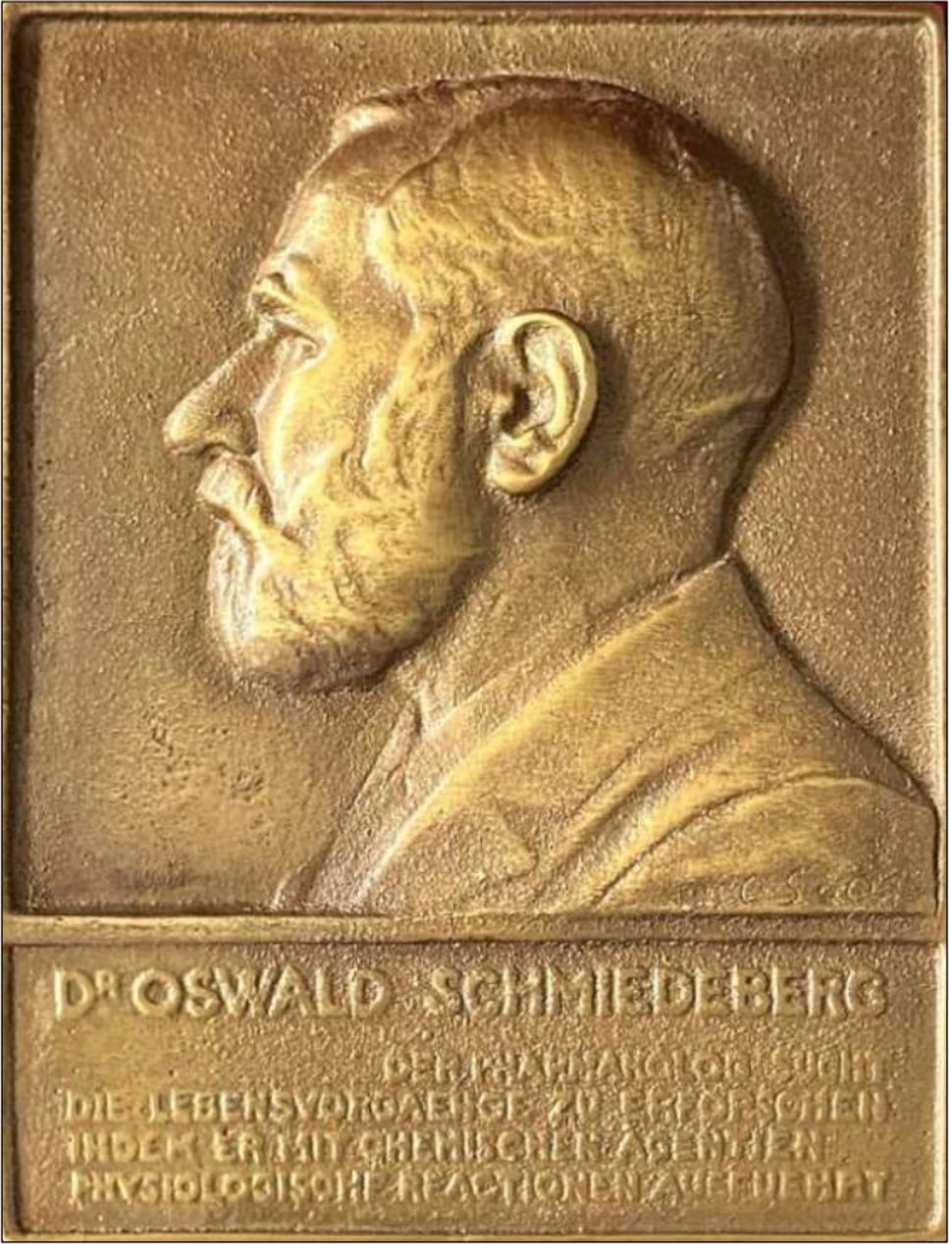
The Schmiedeberg Medal presented to the prize winners. The inscription reads: Dr. Oswald Schmiedeberg Der Pharmakolog Sucht Die Lebensvorgaenge Zu Erforschen Indem Er Mit Chemischen Agentien Physiologische Reactionen Ausfuehrt, which translates to: Dr. Oswald Schmiedeberg the Pharmacologist Seeks to Research Lifes Processes by Performing Physiological Reactions with Chemical Agents (DGPT Archive)

**Fig. 2:**
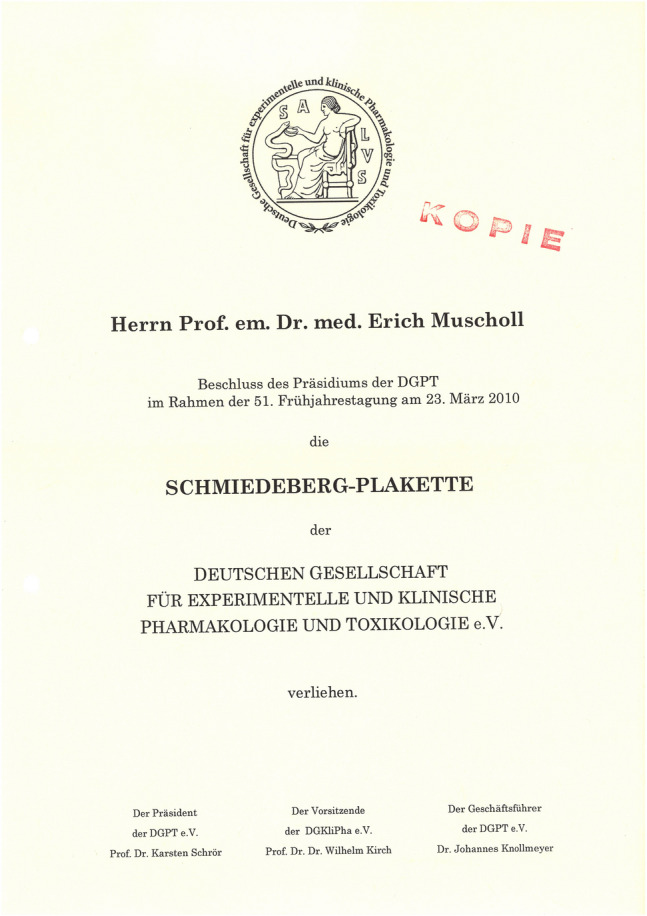
The certificate Erich Muscholl received with his Schmiedeberg Medal in 2010 (ArchMHH Dep. 13 Nr. 13)

Nominations for the Schmiedeberg Medal may be submitted by any of the constituent societies of the DGPT. Each society is entitled to nominate candidates who have demonstrated outstanding scientific achievements in their respective areas of pharmacology or toxicology. All nominations must be accompanied by a comprehensive and well-reasoned justification, clearly outlining the candidate’s scientific contributions and the relevance and significance of their work. In addition to scientific excellence, it is required that the candidate is a role model for junior pharmacologists/toxicologists. Following the submission of nominations, the board of directors of the DGPT conducts a thorough evaluation of each proposal. The assessment focuses on the scientific quality, originality, and impact of the nominee’s work. This process ensures that the Schmiedeberg Medal is awarded only to individuals whose research has resulted in meaningful and lasting contributions to the field. When the award was first established in 1956, a unanimous decision by the board of directors was sufficient to approve a candidate. In 1966, the procedure was amended to require additional confirmation by the General Assembly, with varying majority thresholds. Since 1968 until the mid-1980 s, the confirmation of the Executive Committee’s proposal required a three-quarters majority vote in the General Assembly (Statutes of the German Pharmacological Society (1948–1986): ArchMHH Dep. 13 Nr. 35). For the past decades, nominations have been reviewed by a committee which, after careful consideration, makes the final decision. The committee is composed of the society’s board members (chairpersons and vice-chairpersons of the German Pharmacological Society) (DGP, Deutsche Gesellschaft für Pharmakologie), the German Society for Clinical Pharmacology (DGKliPha, Deutsche Gesellschaft für Klinische Pharmakologie), the German Society for Toxicology (GT, Gesellschaft für Toxikologie), and the Executive Director of the DGPT, all of whom have equal voting rights. The majority of the committee members must vote in favour of a nomination. The nomination and selection procedure for the Schmiedeberg Medal aligns with the procedure for the Fritz-Külz Prize (Halling et al. 2025). The Schmiedeberg Medal is then customarily awarded during the annual DGPT conference, although there have been exceptions for various reasons. The recipient is recognized in a formal award ceremony, during which the Schmiedeberg Medal (Fig. 1) and an official certificate (Fig. 2) are presented. The ceremony also includes a laudatory address that highlights the awardee’s scientific accomplishments and their impact on the field.

To understand the influences that affected the prize winners in their lives, it is necessary to start the observation period with the birth of the oldest award winner, E.P. Pick, who was born in 1872 and end the observation period with the most recent award of our analysis in June 2024 to Franz Hofmann, thus analysing a total of 153 years. During this period there have been diverse influences on the prize winners careers which we studied: As earliest influences on their success, we looked at biographical factors such as geographical and parental origins, gender, education, and the life courses of the prize winners also regarding their career courses. As publications are recognized as the currency of science (Zöllner and Seifert 2024), publishing behaviour is a predictor and confirmation of scientific success. Therefore, we also carried out a bibliometric analysis including the number of publications and co-authors, authorship, journals, language of publication, and the content of research.

## Material and methods

Autobiographies, biographies, curricula vitae, and obituaries were utilized to create an Excel spreadsheet (Table 1) that contains the key data of the prize winners. Many of these documents were accessed from the DGPT’s archive located at the Hannover Medical School (MHH). The archive was compiled and maintained by Erich Muscholl, Schmiedeberg Medal recipient of 2010 (Fig. 2). He also undertook significant work in documenting the history of the DGPT. The specific sources of information for each award winner are listed in Table S1.

To compare the educational and professional backgrounds of the prize winners’ parental home, we categorized the occupations of the parents. We were able to determine the occupation of at least one parent for 42 of the 47 prize winners. These occupations were then classified into the following categories: 0 = unknown, 1 = common labour (e.g. farmworker), 2 = skilled occupation (e.g. businessman), 3 = graduate (e.g. teacher), and 4 = science graduate (e.g. doctor) (Table 1).

For examining the early academic careers of the prize winners, we analysed the subject in which they earned their degrees, excluding those subjects that were started but not completed. Under the category “Science”, we included both “Natural Science” and “Master of Science” degrees (Table 1).

To define the duration of their active scientific career, we measured the time between the first and last publication during their lifetime. Publications that were posthumously published were not included. Our interest then shifted to determining when the Schmiedeberg Medal was awarded in relation to their scientific careers. To do this, we defined the scientific peak based on publication frequency. The year with the highest number of publications, calculated as a 5-year moving average, was considered the career peak.

We accessed life expectancy data for each award winner’s birth year from the Federal Statistical Office (Statistisches Bundesamt) (Sterbefälle und Lebenserwartung. In: Statistisches Bundesamt. https://www.destatis.de/DE/Themen/Gesellschaft-Umwelt/Bevoelkerung/Sterbefaelle-Lebenserwartung/_inhalt.html. Accessed 14 Nov 2023) and compared it to their actual age at death or, if still living, their current age. For consistency, we compared life expectancy for Germany in each birth year, even if the prize winner was born abroad. The primary fields of work of the prize winners were determined from the compiled data in the Excel spreadsheet and categorized using the chapters of the textbook *Basic Knowledge of Pharmacology* (Seifert 2019).

**Fig. 3:**
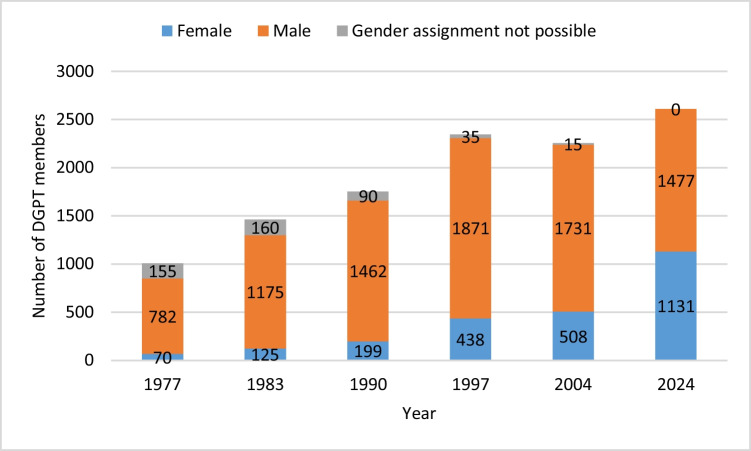
Gender dynamics of the DGPT 1977–2024 shown in absolute number of members. Particulary prior to the year 2000, first names were often abbreviated, making gender assessment impossible in several cases

To analyse the gender development within the pharmacological society, we assessed the member lists of the DGPT, which are found in the DGPT’s archive up to the year 2004. The data for the year 2024 was directly provided by the DGPT. We then sorted the collected lists by gender using first names. The older the member lists were, the more first names were documented only as initials, which meant that no conclusions could be drawn about gender. Until 1977, full first names were documented only occasionally. In the membership list of 1977, there is a notable increase in the number of fully documented names, allowing the majority of first names to be analysed for the first time: 852 of 1007 (84.60%) of the members’ first names could be assigned a gender. This was the first year used in the analysis. The proportion of analysable first names increased until 2004 (the most recent list in the archive) to 99.33% (2239 of 2254 members). The gender distribution of the recent members (2024) was directly provided by the DGPT, without giving out names due to data protection regulations; thus, the gender of all 2608 members was available. As some years were missing from the archive, and assuming that the society’s membership does not change annually, we selected the years 1977, 1983, 1990, 1997, 2004, and 2024 for our analysis. This selection provided a comprehensive view of the gender dynamics within the society over time (Fig. 3).

To analyse the publications of the Schmiedeberg Medal recipients, a comprehensive list of their publications was created by searching the database *Scopus* (http://www.scopus.com), which was the only available resource that covered all the award winners over the necessary time span. The search was conducted using an author-based approach, entering the full last name, full first name, and the initial of any middle name (if applicable), for example, “Heubner Wolfgang” for Wolfgang Heubner and “Pick Ernst P” for Ernst Peter Pick. The search results were carefully screened to remove incorrect or duplicate entries. For example, the search results for “Bock Karl W” (full name: Bock Karl Walter) initially included a publication by “Bock Karl Wilhelm”, which was excluded from the dataset. Similarly, journal names were standardized by correcting spelling mistakes and consolidating variations of a journal’s name over time under its most recent title. For instance, “*Naunyn-Schmiedebergs Archiv für experimentelle Pathologie und Pharmakologie*”, “*Naunyn-Schmiedebergs Archiv für Pharmakologie*”, and “*Naunyn–Schmiedeberg’s Archives of Pharmacology*” were all grouped under the current name “*Naunyn–Schmiedeberg’s Archives of Pharmacology*”.

The resulting publications from all 47 prize winners were transferred into an Excel spreadsheet which then consisted of 9299 publications. Since bibliometrics is only one part of the overall analysis, we focused on a few key variables in the spreadsheet, including: title, author, co-authors, year of publication, journals, document type, and language. In the following step, the data was analysed using IBM SPSS Statistics Version 29.0.0.0 (241) and visualized.

## Results and discussion

### Biographics

#### Country of birth

The majority of prize winners (63.83%, 30 out of 47) were born in Germany, followed by prize winners from England (*n* = 4), Austria (*n* = 3), Sweden and the USA (*n* = 2) and Belgium, Greece, Croatia, the Netherlands, Czech Republic, and Ukraine (*n* = 1 each). This indicates that while the recipient of the Schmiedeberg Medal is expected to be a member of the DGPT, being born outside of Germany does not preclude one from receiving the award (Table 2).

**Table 2:** Countries of birth of the prize winners: by number of prize winners

	Country of birth	Number of prize winners	Percentage of prize winners (%)
1	Germany	30	63.83
2	England	4	8.51
3	Austria	3	6.38
4	Sweden	2	4.26
5	USA	2	4.26
6	Czech Republic	1	2.13
7	Belgium	1	2.13
8	Netherlands	1	2.13
9	Ukraine	1	2.13
10	Greece	1	2.13
11	Croatia	1	2.13
	Total	47	100

#### Parental education

**Fig. 4:**
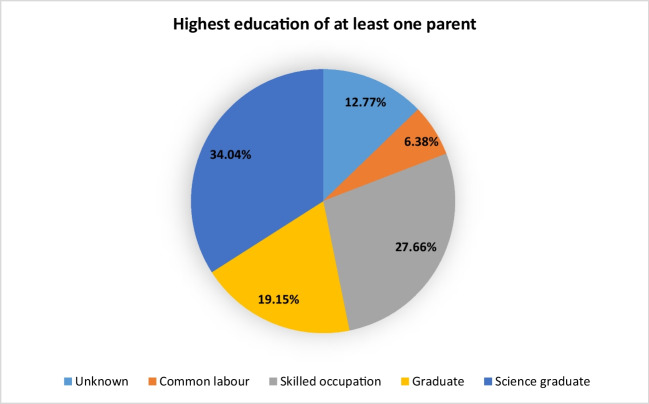
Distribution of the education of the parental home of the prize winners in percent

**Fig. 5:**
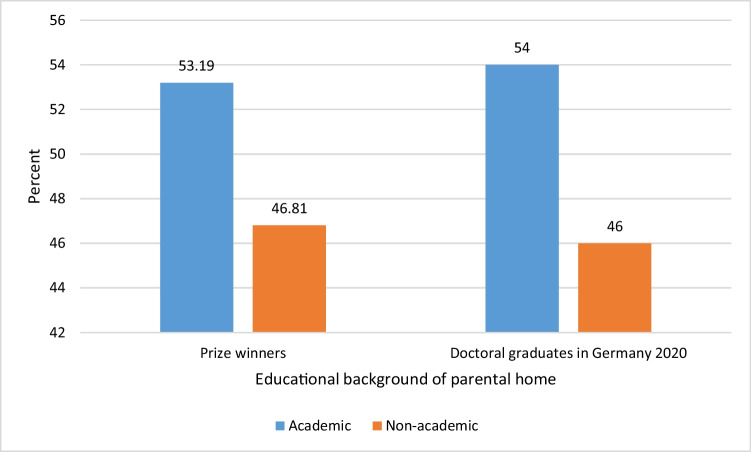
Comparison of the educational background of the Schmiedeberg Medal recipients and doctoral graduates in Germany 2020 (Vom Arbeiterkind zum Doktor | Hochschulbildungsreport. https://www.hochschulbildungsreport2020.de/fokusthemen/arbeiterkinder. Accessed 14 Nov 2024)

More than half (53.19%) of the 47 award winners come from academic households, more than one-third (34.04%) even from academic households with a degree in sciences (Fig. 4). In comparison: in Germany in 2020, 46% of doctoral students came from non-academic households and 54% from academic households (Vom Arbeiterkind zum Doktor | Hochschulbildungsreport. https://www.hochschulbildungsreport2020.de/fokusthemen/arbeiterkinder. Accessed 14 Nov 2024) (Fig. 5).

#### Education

Of the 47 prize winners, 42 (89.36%) had completed a medical degree. Seven of whom also pursued additional qualifications, with four having obtained a degree in pharmacy and three in chemistry. Only five recipients (10.64%) did not study medicine but instead studied science (*n* = 4) and chemistry (*n* = 1) (Table 1). In addition to the field of study, the cities in which the prize winners completed their studies were also examined. This analysis included all cities where the prize winners undertook at least parts of their studies or practical training stages (practical year, internship, medical assistant). A total of 37 cities were identified, with Berlin being the most common. Fourteen prize winners (29.79%) studied in Berlin, followed by Freiburg with 11 prize winners (23.40%), Munich (*n* = 8, 17.02%), Vienna (*n* = 5, 10.64%), and Heidelberg (*n* = 5, 10.64%). Notably, three prize winners studied in five different cities, while 21 individuals remained in a single city for the entirety of their studies (Table 3). This indicates that it was common for the prize winners to relocate during their studies and gain experience at multiple universities. However, this mobility does not appear to be a requirement for academic excellence. Given that most of the award winners are of German origin and the Schmiedeberg Medal is awarded by a German society, it is not surprising that German-speaking cities dominate the list of study locations.

**Table 3:** Cities in which the prize winners have completed at least part of their studies: by number of prize winners

	City of studies	Number of prize winners	Percentage of prize winners (%)
1	Berlin, Germany	14	29.79
2	Freiburg, Germany	11	23.40
3	München, Germany	8	17.02
4	Heidelberg, Germany	5	10.64
5	Wien, Austria	5	10.64
6	Göttingen, Germany	3	6.38
7	Innsbruck, Austria	3	6.38
8	Tübingen, Germany	3	6.38
9	Würzburg, Germany	3	6.38
10	Bonn, Germany	2	4.26
11	Cambridge, Great Britain	2	4.26
12	Düsseldorf, Germany	2	4.26
13	Hamburg, Germany	2	4.26
14	Marburg, Germany	2	4.26
15	Stockholm, Sweden	2	4.26
16	Straßburg, France	2	4.26
17	Athens, Greece	1	2.13
18	Breslau, Poland	1	2.13
19	Gent, Belgium	1	2.13
20	Gießen, Germany	1	2.13
21	Graz, Austria	1	2.13
22	Greifswald, Germany	1	2.13
23	Halle-Wittenberg, Germany	1	2.13
24	Jena, Germany	1	2.13
25	Kiel, Germany	1	2.13
26	Köln, Germany	1	2.13
27	Leipzig, Germany	1	2.13
28	London, Great Britain	1	2.13
29	Mainz, Germany	1	2.13
30	Münster, Germany	1	2.13
31	New York, USA	1	2.13
32	Philadelphia, USA	1	2.13
33	Prag, Czech Republic	1	2.13
34	Rostock, Germany	1	2.13
35	Uppsala, Sweden	1	2.13
36	Utrecht, Netherlands	1	2.13
37	Washington, USA	1	2.13

#### Life expectancy

The prize winners lived to an above-average age throughout the entire observation period. On average, the prize winners lived to be 86.26 years old. Compared to the average life expectancy for their birth cohorts in Germany, the award winners lived on average 32.84 years longer than expected. The oldest prize winner was Marthe Vogt, born in 1903, who passed away just one day after her 100 th birthday in 2003. The life expectancy for her birth cohort (1903) was 53.97 years, meaning she lived 46 years longer than expected. Henry Hallett Dale, on the other hand, had the longest survival relative to his cohort. Born in 1875, he lived to the age of 93, while the life expectancy of his cohort was only 39.96 years; he outlived his cohort by 53.12 years. Notably, no prize winner died before reaching their life expectancy. Manfred Göthert had the smallest increase in life expectancy. He passed away at the age of 79 (79.5) in 2019, while the life expectancy for his birth cohort (1939) was 68.41 years, meaning he lived 11.09 years longer. The prize winners who are still alive today have exceeded their life expectancy by at least a decade so far (Table 4).

**Table 4:** Life data of the prize winners: by age of death

	Name of prize winner	Age at death/*current age (12/2024) (years)	Life expectancy in Germany (years)	Survival past life expectancy (years)
1	Vogt	100	53.97	46.03
2	Remmer	98.16	49	49.16
3	Brock	96	53.96	42.04
4	Schmidt	94.66	44.29	50.37
5	Hornykiewicz	93.5	61.61	31.89
6	Kosterlitz	93.41	47.85	45.56
7	Blaschko	93.25	46.32	46.93
8	Dale	93.08	39.96	53.12
9	Feldberg	92.91	46.32	46.59
10	Axelrod	92.5	53.96	38.54
11	Muscholl	92.5	61.61	30.89
12	Mutschler*	93.41	64.56	28.85
13	Lembeck	92.25	58.58	33.67
14	Philippu*	93.08	64.56	28.52
15	Herken	90.75	53.96	36.79
16	Vater	89.75	54.87	34.88
17	Burn	89.33	44.07	45.26
18	Kuschinsky	88.83	48.45	40.38
19	Loewi	88.5	39.48	49.02
20	Muschaweck	88.33	54.7	33.63
21	Bock*	89.16	66.84	22.32
22	Reuter	87.83	66.69	21.14
23	Pick	87.58	39.28	48.3
24	Schulemann	87.08	43.07	44.01
25	Markwardt	86.75	60.61	26.14
26	Buelbring	86.5	53.97	32.53
27	Starke	86.08	67.83	18.25
28	Schaumann	85.75	43.86	41.89
29	Schultz	85.5	67.34	18.16
30	Ariëns	84.08	54.7	29.38
31	Trendelenburg	83.83	58.58	25.25
32	Eichelbaum*	83.41	68.86	14.55
33	Krayer	82.33	45.86	36.47
34	Liljestrand	81.75	42.47	39.28
35	Brodie	81.5	50.24	31.26
36	Hofmann*	82.5	69.14	13.36
37	Schroer*	82.5	69.14	13.36
38	Heubner	79.66	40.35	39.31
39	Göthert	79.5	68.41	11.09
40	v. Euler	78.08	49	29.08
41	Schild	78	49.53	28.47
42	Heymans	76.25	44.07	32.18
43	Dost	75.25	52.06	23.19
44	Fleckenstein	74.91	54.87	20.04
45	Verney	72.91	44.48	28.43
46	Wilbrandt	72.5	50.24	22.26
47	Holtz	68.75	47.29	21.46
	∅	86.26	53.42	32.84

*Prize winner is still alive in 12/2024

**Fig. 6:**
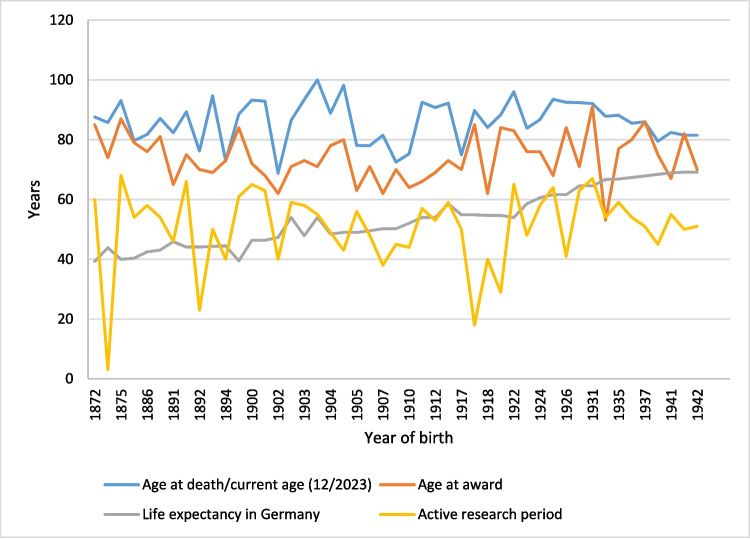
Overview over the life and career courses of the prize winners: by year of birth

Although life expectancy has steadily increased over the last century, the age at death for the award winners has remained relatively constant, meaning that relative survival has decreased over time (Table 4, Fig. 6).

The primary factors behind the increase in life expectancy over the past century include lower infant and child mortality rates, as well as improvements in the quality of life and healthcare (Sterbefälle und Lebenserwartung. In: Statistisches Bundesamt. https://www.destatis.de/DE/Themen/Gesellschaft-Umwelt/Bevoelkerung/Sterbefaelle-Lebenserwartung/_inhalt.html. Accessed 14 Nov 2024).

Our study highlights the similarities among the Schmiedeberg Medal winners, all of whom enjoyed a high quality of life and education, contributing to a longer life expectancy even more than a century ago. The positive correlation between education and life expectancy has been well-documented in previous studies, although the causal relationship remains multifactorial (Günther and Huebener 2018).

#### Career

We then examined the duration of the award winners’ active scientific careers. Our objective was to determine whether this duration has increased over time, in line with the general rise in life expectancy over the past century (Sterbefälle und Lebenserwartung. In: Statistisches Bundesamt. https://www.destatis.de/DE/Themen/Gesellschaft-Umwelt/Bevoelkerung/Sterbefaelle-Lebenserwartung/_inhalt.html. Accessed 14 Nov 2024). On average, the prize winners published for 50.57 years (ranging from 3 to 68 years) (Table 5). This figure has remained relatively stable over time, reflecting the consistent age and life expectancy of the prize winners. While overall life expectancy has significantly increased over the last century, the life expectancy of the prize winners has consistently remained above average. From the peak of their careers, it took an average of 20.74 years for the Schmiedeberg Medal to be awarded, with recipients receiving the honour at an average age of 73.85 (Table 5). The latency from career peak to award has remained relatively stable over the observation period (Table S2, Fig. S1). In one instance, the award was given to the prize winner (H. Kosterlitz) 3 years before his career peak. E.P. Pick experienced the longest wait; his highest 5-year publication average occurred in 1911, yet the Schmiedeberg Medal was not awarded to him until 46 years later in 1957, 4 years after his last publication, and 3 years before his death. R. Muschaweck had an even longer wait, receiving the award at the age of 51. However, given that only seven of his publications are included in the database, we question the significance of the career peak by publication count in this case. On average, the Schmiedeberg Medal was awarded 12.61 years before the prize winner’s death (ranging from 0 to 34.83 years).

**Table 5:** Overview of the main career data of the prize winners: by latency from career peak to award of the Schmiedeberg Medal

	Name of prize winner	Latency from career peak to award (years)	Active research period (years)	Career peak (year)	Age at award (years)
1	Muschaweck	51	29	1951	80
2	Pick	46	60	1911	83
3	Muscholl	43	41	1967	70
4	Verney	41	40	1926	91
5	Dale	40	68	1922	65
6	Schaumann	34	3	1931	76
7	Brock	32	65	1963	70
8	Loewi	32	61	1925	69
9	Heubner	32	54	1924	81
10	Schulemann	31	54	1938	67
11	Heymans	31	23	1931	72
12	Trendelenburg	30	48	1968	70
13	Starke	28	51	1995	77
14	Buelbring	27	59	1947	69
15	Schild	26	48	1951	71
16	Philippu	25	67	1997	68
17	Kuschinsky	24	49	1958	71
18	Göthert	24	45	1990	68
19	Schultz	23	54	1993	84
20	Wilbrandt	23	45	1954	82
21	Schroer	22	51	1990	84
22	Vater	21	18	1981	80
23	Hofmann	20	50	2004	71
24	Bock	19	59	1993	87
25	Reuter	19	54	1968	76
26	Herken	18	53	1963	62
27	Feldberg	16	63	1952	75
28	Krayer	16	46	1948	70
29	Dost	16	44	1958	74
30	Blaschko	15	65	1957	85
31	v. Euler	15	56	1953	75
32	Holtz	14	40	1950	64
33	Lembeck	13	59	1982	66
34	Fleckenstein	13	50	1974	63
35	Markwardt	12	58	1988	78
36	Axelrod	12	57	1966	84
37	Ariëns	12	40	1968	79
38	Mutschler	10	63	1992	53
39	Remmer	10	43	1975	73
40	Brodie	10	38	1959	76
41	Liljestrand	9	58	1953	62
42	Burn	8	66	1959	62
43	Eichelbaum	5	55	2003	73
44	Hornykiewicz	4	64	1990	71
45	Vogt	4	55	1970	86
46	Schmidt	2	50	1960	85
47	Kosterlitz	−3	58	1979	73
	∅	20.74	50.57		73.85

A closer examination of the four Nobel Prize laureates among the recipients of the Schmiedeberg Medal reveals: Otto Loewi and Henry Dale were awarded the Nobel Prize in 1936, yet they received the Schmiedeberg Medal only much later—Loewi after 21 years (1957) and Dale after 26 years (1962). In 1970, the Nobel Prize was jointly awarded to Ulf von Euler and Julius Axelrod. Notably, Axelrod followed a similar trajectory as the 1936 prize winners, receiving the Nobel Prize prior to being honoured with the Schmiedeberg Medal in 1978. In contrast, Ulf von Euler was awarded the Schmiedeberg Medal in 1968, 2 years before receiving the Nobel Prize. This suggests that the DGPT’s selection process operates independently of the Nobel Committee and, in the case of von Euler, recognized his contributions at a slightly earlier stage.

#### Gender

Among the 47 prize winners, only 2 were women, accounting for just 4.26%. The two female prize winners were Marthe Louise Vogt and Edith Bülbring. Both were born in Germany in 1903 and fled to London due to the upheaval caused by the Second World War—Bülbring was of Jewish descent, and Vogt came from a family opposed to the Nazi regime. Bülbring’s primary place of work was Oxford, where she became a professor and worked until her death in 1990. Vogt moved to England, where she worked in Edinburgh and Cambridge, and later relocated to California in her 90 s due to health reasons, where she passed away in 2003. Both women were pivotal in shaping British pharmacology in the post-war period. They were both awarded the Schmiedeberg Medal in 1974 (Löffelholz and Trendelenburg 2008; Philippu 2017). Although the proportion of women in the medical field has steadily increased over the years (Frauenanteil Professuren—https://www.innovative-frauen-im-fokus.de/infopool/daten-und-fakten/frauen-in-der-wissenschaft/frauenanteil-in-professuren-zeitreihe/. Accessed 16 Dec 2024; Studierende insgesamt und Studierende Deutsche im Studienfach Medizin (Allgemein-Medizin) nach Geschlecht. In: Statistisches Bundesamt. https://www.destatis.de/DE/Themen/Gesellschaft-Umwelt/Bildung-Forschung-Kultur/Hochschulen/Tabellen/lrbil05.html. Accessed 16 Dec 2024), and the percentage of women in pharmacology (as reflected by the gender distribution of DGPT members) has steadily risen from 8.2% in 1977 to 43.37% in 2024, no women have been awarded the Schmiedeberg Medal since 1974.

When examining the top scientists in the field, the underrepresentation of women persists. Of the 42 DGPT members recognized among the “best scientists” in biology and biochemistry on www.research.com in 2022, only two were women, accounting for just 4.76% (Fox and Seifert 2024). This indicates that while the representation of women among physicians and pharmacologists is improving, a disparity remains between the number of women in the field on one hand and leading scientists and those honoured with the Schmiedeberg Medal on the other hand. This issue is not unique to pharmacology but is part of a broader phenomenon known as the “gender award gap” across various disciplines (Halling et al. 2022; Meho 2021).

**Fig. 7:**
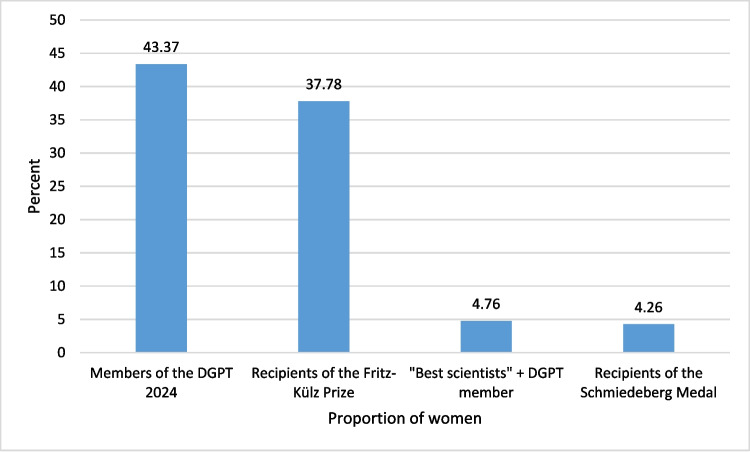
Proportion of women among different scientific groups in percent (Halling et al. 2025) (Fox and Seifert 2024)

To assess whether the DGPT discriminates against women in awarding scientific prizes, one can look at the Fritz-Külz Prize, granted by the DGPT to early-career scientists, typically based on their doctoral theses. Of the 45 recipients of this prize so far, 28 were male (62.22%), and 17 were female (37.78%), with the proportion of women increasing over time (Halling et al. 2025). This suggests that female scientists are in fact being recognized and promoted by the DGPT, with the gender award gap slowly narrowing for emerging scientists. However, it remains prevalent for those who are honoured for their lifetime achievements. This is, in part, explained by the typical latency between a scientist’s accomplishments and the awarding of such honours as the Schmiedeberg Medal. Given the growing number of accomplished female pharmacologists—as reflected, for example, in the increasing recognition of women through the Fritz-Külz Prize—it is reasonable to expect that female scientists will be more prominently represented among future recipients of the Schmiedeberg Medal. The distribution of women across the various groups is depicted in Fig. 7.

The gender award gap can also be observed in the most prestigious scientific prize: the Nobel Prize. Of the 55 laureates from 2006 to 2022 in the categories related to pharmacology, “physiology or medicine” and “chemistry”, 10 were women (18.18%) and 45 were men (81.82%) (Bünemann and Seifert 2024).

### Bibliometrics

#### Publications

**Fig. 8:**
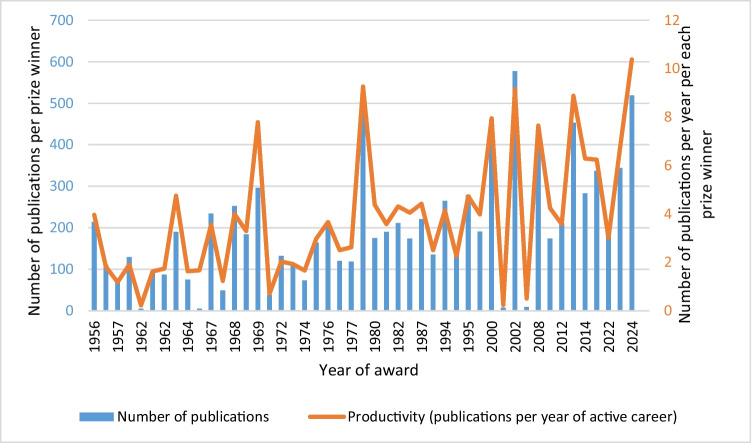
Course of number of publications and productivity (publications per year of active career) of each prize winner: by year of award

The publications by the prize winners analyzed in this study were released between the years of 1893 and 2023. On average, the prize winners published 201.07 publication during their active careers (Table 6). This was not constant over time. The absolute number of publications per prize winner increased during the observation period, as illustrated in Fig. 8 (Table 6).

**Table 6:** Number of publications of each prize winner, by absolute number of publications (Source: www.scopus.com)

	Name of prize winner	Overall number of publications	Year of award
1	Mutschler	577	2002
2	Axelrod	528	1978
3	Hofmann	519	2024
4	Markwardt	461	2000
5	Schroer	453	2012
6	Eichelbaum	421	2008
7	Starke	344	2023
8	Schultz	337	2016
9	Brodie	296	1969
10	Göthert	283	2014
11	Lembeck	279	1995
12	Hornykiewicz	265	1994
13	Feldberg	252	1968
14	Burn	234	1967
15	Fleckenstein	221	1987
16	Heubner	214	1956
17	Kosterlitz	212	1976
19	Bock	211	2012
18	Kuschinsky	211	1982
20	Philippu	202	2022
21	Trendelenburg	191	1998
23	Herken	190	1981
22	Holtz	190	1964
24	v. Euler	184	1968
25	Ariëns	175	1980
27	Muscholl	174	2010
26	Remmer	174	1985
28	Vogt	164	1974
29	Brock	146	1995
30	Reuter	135	1987
31	Blaschko	132	1972
32	Dale	129	1962
33	Schild	120	1977
34	Wilbrandt	118	1977
35	Buelbring	114	1974
36	Loewi	111	1957
37	Liljestrand	94	1962
38	Schmidt	87	1962
39	Krayer	75	1964
40	Dost	73	1974
41	Pick	71	1957
42	Verney	49	1967
43	Schulemann	38	1969
44	Vater	9	2002
45	Muschaweck	7	2002
46	Heymans	5	1962
47	Schaumann	5	1965
	∅	201.07

In addition, we assessed productivity by measuring the number of publications per year of active career. Our analysis revealed that not only the absolute number of publications but also productivity (publications per year of active career) increased over time, indicating that the prize winners became more productive during the observation period. On average, the prize winners published 3.85 papers per year of active career, with the latest analysed prize winner F. Hofmann (2024) being the most productive, with 10.38 publications per year (Table 7).

**Table 7:** Productivity (publications per year of active career) of each prize winner, by average productivity

	Name of prize winner	Number of publications per year of active career	Year of award
1	Hofmann	10.38	2024
2	Axelrod	9.26	1978
3	Mutschler	9.16	2002
4	Schroer	8.88	2012
5	Markwardt	7.95	2000
6	Brodie	7.79	1969
7	Eichelbaum	7.65	2008
8	Starke	6.75	2023
9	Göthert	6.29	2014
10	Schultz	6.24	2016
11	Holtz	4.75	1964
12	Lembeck	4.73	1995
13	Fleckenstein	4.42	1987
14	Ariëns	4.38	1980
15	Kuschinsky	4.31	1982
16	Muscholl	4.24	2010
17	Hornykiewicz	4.14	1994
18	Remmer	4.05	1985
19	Feldberg	4.00	1968
20	Trendelenburg	3.98	1998
21	Heubner	3.96	1956
22	Kosterlitz	3.66	1976
23	Bock	3.58	2012
24	Herken	3.58	1981
25	Burn	3.55	1967
26	v.Euler	3.29	1968
27	Philippu	3.01	2022
28	Vogt	2.98	1974
29	Wilbrandt	2.62	1977
30	Reuter	2.50	1987
31	Schild	2.50	1977
32	Brock	2.25	1995
33	Blaschko	2.03	1972
34	Buelbring	1.93	1974
35	Dale	1.90	1962
36	Loewi	1.82	1957
37	Schmidt	1.74	1962
38	Schaumann	1.67	1965
39	Dost	1.66	1974
40	Krayer	1.63	1964
41	Liljestrand	1.62	1962
42	Verney	1.23	1967
43	Pick	1.18	1957
44	Schulemann	0.70	1969
45	Vater	0.50	2002
46	Muschaweck	0.24	2002
47	Heymans	0.22	1962
	∅	3.85	

A linear regression analysis of both the total number of publications over time and publications per active year showed a significant positive correlation to the year of the award. Later award winners, therefore, have higher productivity (Table S3, Fig. S2, Table S4, Fig. S3). For a useful comparison, the data can be contrasted with the 2023 “barometer of science” (Fabian et al. 2024) which examined the publication behaviours of German researchers, including the annual number of publications by field of study and career stage (“Prof”, “Juniorpof”, “Postdoc”, “Prädoc”). In the natural sciences (the field in which pharmacology is represented), the group of professors published the most, with an average of 6.8 publications per year, while “Prädocs” published the least, at 0.4 publications per year. When averaging across all career stages, researchers published an average of 3.48 publications per year in 2023. We applied a similar methodology to our analysis of Schmiedeberg Medal recipients, by incorporating publications from all stages of their careers. When comparing the 3.48 publications of German scientists in 2023 to the 3.85 publications of the Schmiedeberg prize winners, it is evident that the latter were slightly more productive on average, even when accounting for periods of lower productivity, such as those early in their careers or at the start of the observation period.

**Fig. 9:**
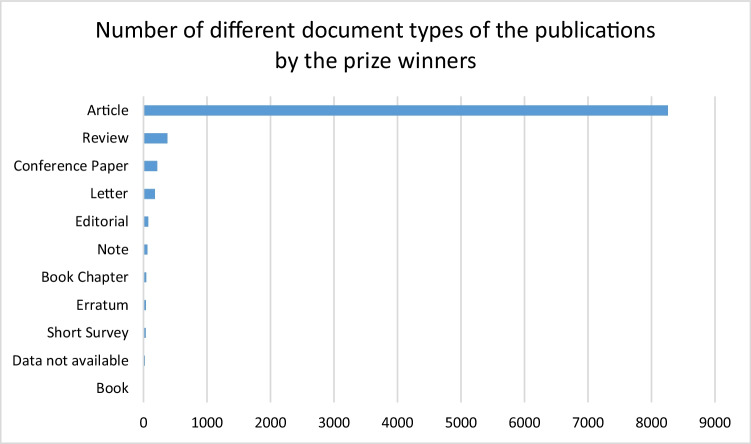
Distribution of document types of the publications of the prize-winners in absolute numbers

**Fig. 10:**
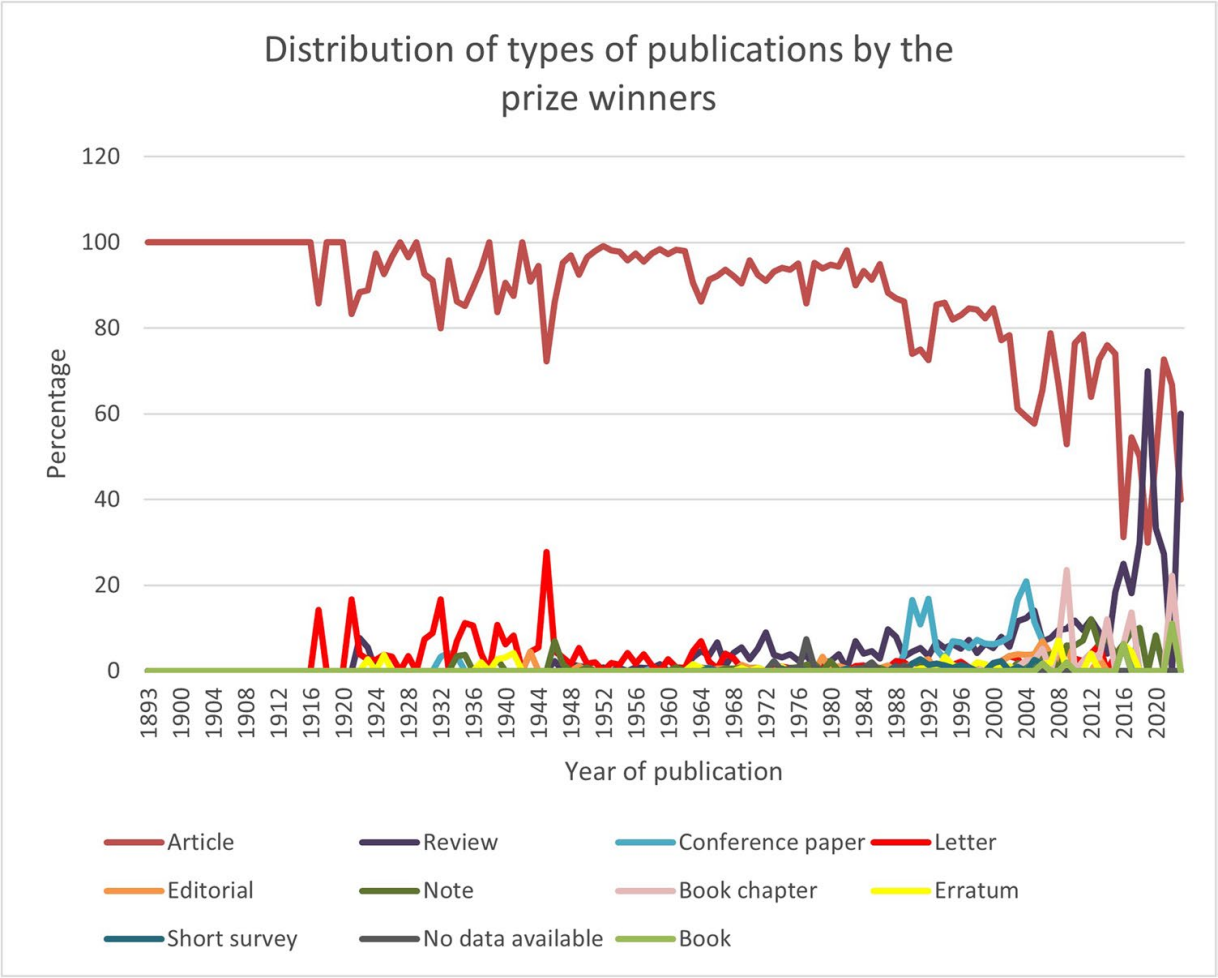
Course of the document types of publications by the prize winners: by year of publication

An analysis of the publication types reveals that the “Article” was by far the most common document type, clearly dominating the landscape with overall 88.78% of all publications. At the beginning of the last century, “Letters” were also relatively prevalent. However, towards the end of the century, other document types began to gain prominence, with a particularly noticeable increase in the number of “Reviews” which overall contributes to 4.04% of all publications (Fig. 9, Table S5). This illustrates that expert evaluation and peer assessment control is gaining importance. The evolution of the distribution of these document types is illustrated in Fig. 10.

#### Co-authors

Not only has the number of publications increased over time, but the number of co-authors has also risen, indicating a growing trend of collaboration within scientific teams throughout the observation period. In total, 7830 co-authors contributed to the 9299 publications. The number of co-authors with whom each individual prize winner collaborated varies significantly. F. Hofmann tops the list with 1356 co-authors, having worked with as many as 53 co-authors on a single publication (Eden et al. 2016). On average, each award winner collaborated with 178 co-authors (ranging from 1 to 1356) over the course of their career (Table 8, Table S6, Fig. S4).

**Table 8:** Number of co-authors of each prize winner, by absolute number

	Name of prize winner	Number of co-authors
1	Hofmann	1356
2	Eichelbaum	950
3	Mutschler	702
4	Schroer	518
5	Axelrod	450
6	Hornykiewicz	412
7	Schultz	356
8	Göthert	278
9	Markwardt	255
10	Brodie	253
11	Bock	238
12	Starke	174
13	Lembeck	167
14	Fleckenstein	158
15	Brock	127
16	Remmer	124
17	Feldberg	115
18	Philippu	111
19	Kosterlitz	100
20	Trendelenburg	100
21	Burn	99
22	Herken	95
23	Heubner	92
24	Reuter	91
25	Blaschko	90
26	v. Euler	83
27	Holtz	78
28	Muscholl	71
29	Ariëns	68
30	Dale	68
31	Kuschinsky	61
32	Vogt	59
33	Schild	55
34	Wilbrandt	53
35	Buelbring	48
36	Krayer	47
37	Schmidt	46
38	Verney	43
39	Liljestrand	39
40	Pick	27
41	Loewi	25
42	Dost	25
43	Vater	19
44	Schulemann	18
45	Muschaweck	10
46	Heymans	3
47	Schaumann	1
	∅	177.83

**Fig. 11:**
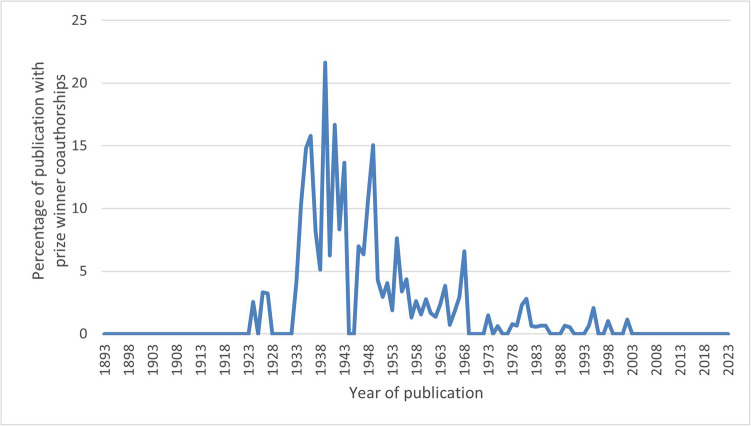
Percentage of publications co-authored with other awardees relative to the total number of publications per year, by year of publication

An analysis of the collaborative networks among Schmiedeberg Medal recipients was performed. Although co-authored publications represent only a small proportion of the total scientific output—181 publications, accounting for just 1.95% of the overall total—there was a noticeable concentration of such collaborations in the mid-twentieth century. Since that time, however, there has been a consistent and noticeable decline in collaborations among awardees. This decline may be indicative of the broader internationalization of scientific research, where collaborations are no longer confined to traditional academic or national networks that are often referred to as certain “schools”. Today, researchers are increasingly globally connected, fostering a more diverse range of international and interdisciplinary collaborations. The most frequent collaboration among Schmiedeberg Medal recipients was between Axelrod and Brodie, who co-authored 25 publications together. Following closely were Burn and Bülbring, with 24 joint publications. The prize winner with the highest number of publications involving other recipients is Gustav Kuschinsky, who contributed to 37 publications co-authored with 4 other prize winners: Mutschler, Reuter, Muscholl, and Trendelenburg (Table S7). The percentage of publications co-authored with other awardees relative to the total number of publications per year is shown in Fig. 11.

#### Authorship

The distinction between first and senior authorship is clearly outlined in the MHH’s rules of good scientific practice: “First author/last author: In principle, the rule applies that the author who writes a manuscript for publication can also claim first authorship. (…) The last position in a publication, the senior authorship, is usually held by the person responsible for the project, (…). The person responsible for the project is the person who initiated the project on which the publication is based, who participated actively or in an advisory capacity in the implementation of the project and who supported the project itself with advice and ideas based on their experience. (…)” (https://www.mhh.de/fileadmin/mhh/hannover-biomedical-research-school/HBRS/Downloads/GSP_Principles_of_MHH___Version_5.0__2022_.pdf. Accessed 26 Dec 2024). In summary, the senior author is typically the leader of the research group, overseeing the entire project.

**Fig. 12:**
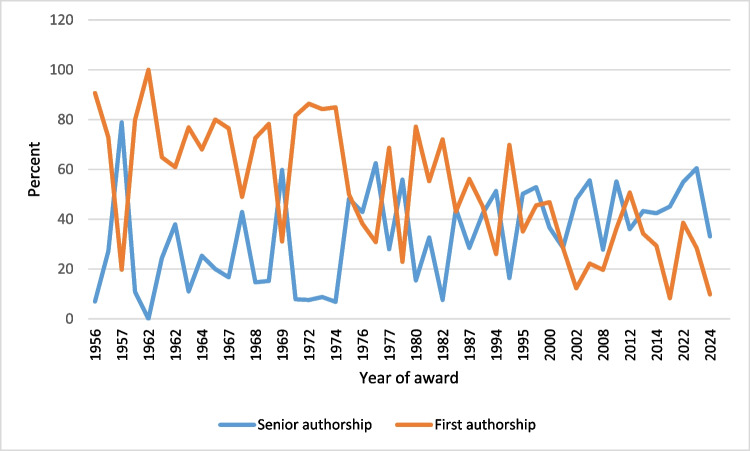
Proportions of first and senior authorships of each prize winner in percent: by year of award

Among the Schmiedeberg Medal recipients, 52.33% of their publications listed them as the first author (including sole authorships) (Table 9), while in 33.38% of their publications, they were listed as the last author (Table 10). Over the course of the observation period, there was a noticeable shift towards senior authorship and a decline in first authorships, as shown in Fig. 12.

**Table 9:** Percentage of first authorships and absolute numbers of publications as first author of each prize winner, by percentage of first authorship

	Name of prize winner	First authorship (%)	Number of publications as first author
1	Heymans	100	5
2	Heubner	90.65	194
3	Blaschko	86.36	114
4	Dost	84.93	62
5	Buelbring	84.21	96
6	Schulemann	81.58	31
7	Schaumann	80	4
8	Dale	79.84	103
9	v. Euler	78.26	144
10	Ariëns	77.14	135
11	Holtz	76.84	146
12	Burn	76.5	179
13	Loewi	72.97	81
14	Feldberg	72.62	183
15	Kuschinsky	72.04	152
16	Brock	69.86	102
17	Wilbrandt	68.64	81
18	Krayer	68	51
19	Liljestrand	64.89	61
20	Schmidt	60.92	53
21	Fleckenstein	56.11	124
22	Herken	55.26	105
23	Bock	50.71	107
24	Vogt	50	82
25	Verney	48.98	24
26	Markwardt	46.85	216
27	Trendelenburg	45.55	87
28	Reuter	45.19	61
29	Remmer	43.1	75
30	Philippu	38.61	78
31	Kosterlitz	38.21	81
32	Muscholl	36.21	63
33	Lembeck	35.13	98
34	Schroer	34.22	155
35	Brodie	31.08	92
36	Schild	30.83	37
37	Göthert	29.33	83
38	Muschaweck	28.57	2
39	Starke	28.49	98
40	Hornykiewicz	26.04	69
41	Axelrod	22.92	121
42	Vater	22.22	2
43	Pick	19.72	14
44	Eichelbaum	19.71	83
45	Mutschler	12.31	71
46	Hofmann	9.83	51
47	Schultz	8.31	28
	∅	52.33

**Table 10:** Percentage of senior authorships and absolute numbers of publications as senior author of each prize winner, by percentage of senior authorship

	Name of prize winner	Senior authorship (%)	Number of publications as senior author
1	Pick	78.87	56
2	Schild	62.5	75
3	Starke	60.47	208
4	Brodie	59.8	177
5	Axelrod	55.87	295
6	Vater	55.56	5
7	Muscholl	55.17	96
8	Philippu	54.95	111
9	Trendelenburg	52.88	101
10	Hornykiewicz	51.32	136
11	Lembeck	50.18	140
12	Vogt	48.17	79
13	Mutschler	48.01	277
14	Schultz	45.1	152
15	Remmer	44.25	77
16	Schroer	43.27	196
17	Kosterlitz	42.92	91
18	Verney	42.86	21
19	Göthert	42.4	120
20	Reuter	42.22	57
21	Schmidt	37.93	33
22	Markwardt	36.66	169
23	Bock	36.02	76
24	Hofmann	33.14	172
25	Herken	32.63	62
26	Muschaweck	28.57	2
27	Fleckenstein	28.51	63
28	Wilbrandt	27.97	33
29	Eichelbaum	27.79	117
30	Loewi	27.03	30
31	Krayer	25.33	19
32	Liljestrand	24.47	23
33	Schaumann	20	1
34	Burn	16.67	39
35	Brock	16.44	24
36	Ariëns	15.43	27
37	v. Euler	15.22	28
38	Feldberg	14.68	37
39	Holtz	11.05	21
40	Dale	10.85	14
41	Buelbring	8.77	10
42	Schulemann	7.89	3
43	Blaschko	7.58	10
44	Kuschinsky	7.58	16
45	Heubner	7.01	15
46	Dost	6.85	5
47	Heymans	0	0
	∅	33.38

The rise in the number of publications and co-authors, alongside the shift towards senior authorship, highlights the growing trend of collaboration in (international) research teams and the increasing interconnectedness of the scientific community. This underscores the importance of networks in today’s research landscape (Ärzteblatt DÄG Redaktion Deutsches, 2008. Gemeinsam veröffentlichen oder untergehen. In: Deutsches Ärzteblatt. https://www.aerzteblatt.de/archiv/60176/Gemeinsam-veroeffentlichen-oder-untergehen. Accessed 17 Dec 2024).

#### Journals: the importance of Naunyn–Schmiedeberg’s Archives of Pharmacology

A total of 9299 papers were published in 1053 different journals, with a distinctly skewed distribution. Nearly 50% of the papers (49.79%) were published in just 1.99% of the journals (*n* = 21), while 28.8% of the papers appeared in only 0.47% of journals (*n* = 5). The *Naunyn–Schmiedeberg’s Archives of Pharmacology* emerged as the journal with the highest number of publications, contributing 15.87% (*n* = 1476) of the total publications. It was followed by the *Journal of Physiology* (5.1%; *n* = 474) and the *Journal of Pharmacology and Experimental Therapeutics* (2.9%; *n* = 266). Notably, in 519 journals (49.29%), only 1 publication by a Schmiedeberg Medal recipient was recorded. The 21 most significant journals are listed in Table 11.

**Table 11:** The 21 most frequently used journals for publication of the prize winners, by absolute number of publications

	Name of journal	Number of publications	Percent (%)	Cumulative percent (%)
1	Naunyn–Schmiedeberg’s Archives of Pharmacology	1476	15.87	15.87
2	The Journal of Physiology	474	5.10	20.97
3	The Journal of Pharmacology and Experimental Therapeutics	266	2.86	23.83
4	Klinische Wochenschrift	238	2.56	26.39
5	British Journal of Pharmacology	224	2.41	28.8
6	Arzneimittel-Forschung	210	2.26	31.06
7	Pflugers Archiv European Journal of Physiology	192	2.06	33.12
8	European Journal of Pharmacology	173	1.86	34.98
9	Biochemical Pharmacology	155	1.67	36.65
10	Nature	150	1.61	38.26
11	British Journal of Pharmacology and Chemotherapy	135	1.45	39.71
12	The Journal of Biological Chemistry	130	1.40	41.11
13	Deutsche Medizinische Wochenschrift	112	1.20	42.32
14	Proceedings of the National Academy of Sciences of the United States of America	104	1.12	43.43
15	British Medical Journal	98	1.05	44.49
16	Life Sciences	93	1.00	45.49
17	Science	86	0.92	46.41
18	Die Naturwissenschaften	85	0.91	47.33
19	Experientia	78	0.84	48.17
20	European Journal of Clinical Pharmacology	77	0.83	48.99
21	FEBS Letters	74	0.80	49.79

The fact that the *Journal of Physiology* holds the second position further highlights the deep-rooted relationship between physiology and pharmacology, with pharmacology having originally emerged from the field of physiology. This enduring connection is also immortalized in the inscription on the Schmiedeberg Medal itself (Fig. 1).

**Fig. 13:**
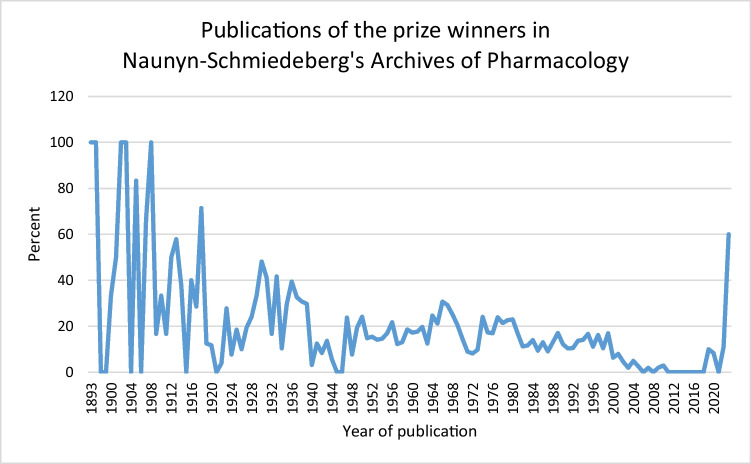
Percentage of publications of the prize winners in NSAP, by year of publication

The importance of Naunyn*–Schmiedeberg’s Archives of Pharmacology* over time is illustrated in Fig. 13 which shows that the proportion of publications in the NSAP, although it is the most important single journal, is decreasing (Fig. 13) There were no publications immediately after the Second World War, as the NSAP was subject to a forced break after World War II (Starke 1998).

**Fig. 14:**
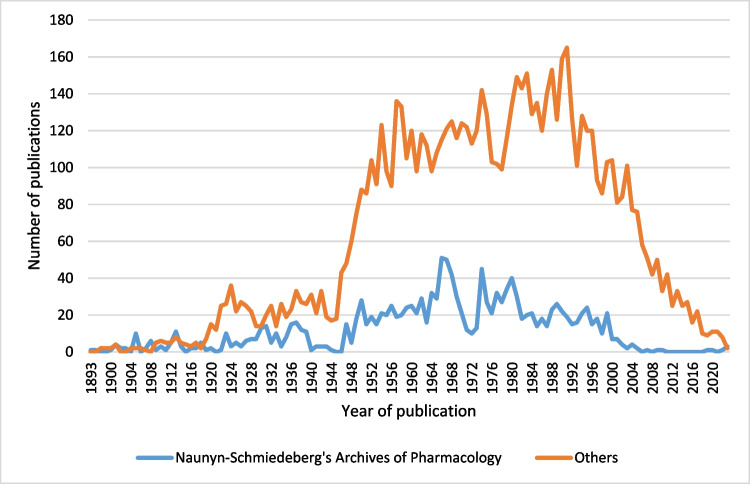
Absolute number of publications in the NSAP compared to the number of publications in all other journals combined, by year of publication

In absolute terms, the peak of publications by the prize winners in the NSAP occurred in 1966, with 51 publications, accounting for 30.72% of total publications that year. Subsequently, the number of publications in the NSAP steadily decreased, with a complete absence of publications by Schmiedeberg Medal recipients between 2011 and 2018. However, recent years might show signs of recovery, albeit with relatively low absolute figures. In 2022, one out of nine publications appeared in the NSAP (11.11%), while in 2023, three out of five publications were published there (60%). It should be noted, however, that all publications in the NSAP in 2022 and 2023 were authored by A. Philipppu. The true value of the journals revival is therefore questionable - nevertheless, it appears to be receiving increased attention. In Fig. 14 there appears to be a noticeable decline in the number of publications in recent years. However, this is due to the fact, that future Schmiedeberg Medal recipients-who have not yet been identified or included in the analysis-are already publishing and have been active in recent years. Their publications have not yet been accounted for. The apparent decline is therefore a result of the latency betwen the publication of their work and the later recognition of them as Schmiedeberg Medal recipients. When looking at the individual prize winner, however, there is a clear increase in both the number of publications and the productivity per prize winner (Fig. 8).

#### Language

**Fig. 15:**
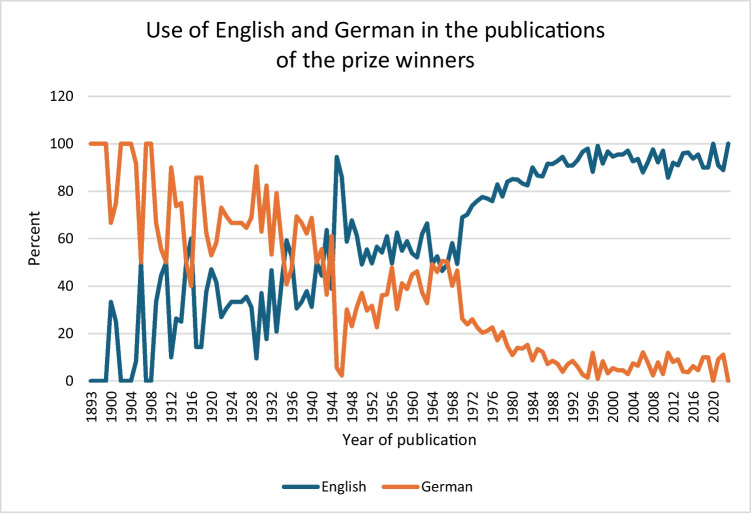
Comparison of the use of English and German in original publications in percent by year of publication

A total of 14 languages were used in the original publications (Table S9) The most frequently used language is English, accounting for 72.28% (*n* = 6721) of the publications, followed by German with 25.2% (*n* = 2346). In 0.9% of cases (*n* = 87), the original language was not specified. Given that English and German are the predominant languages, we also examined the trend in the use of these two languages by the prize winners over time. There has been a continuous increase in the use of English and a corresponding decrease in the use of German. Immediately following the end of World War II, there was a decline in German publications, which then rose again to pre-war levels. One notable point is in 1973, when a clear divergence between the two curves is observed (Fig. 15). This shift coincides with the change in 1973, when the *Naunyn–Schmiedeberg’s Archives of Pharmacology* made English the mandatory language for publication (Starke 1998).

#### Research

**Fig. 16:**
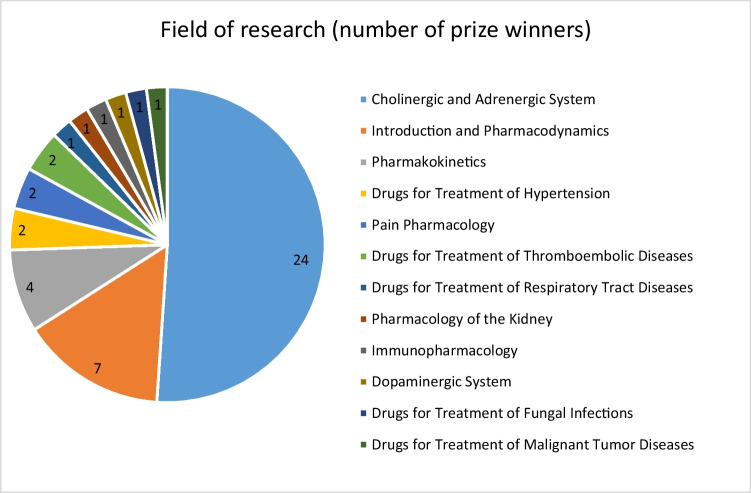
Distribution of the main fields of research of the prize winners categorized based on the chapters of the textbook “Basic Knowledge of Pharmacology” (Seifert 2019), in absolute numbers of prize winners

When examining the content of the research, one topic stands out: 51.06% of the prize winners worked on the “Cholinergic and Adrenergic Systems”, a trend that remained stable throughout the entire observation period. The remaining 48.94% of the awardees worked on 11 other topics, with the most prominent being “Introduction and Pharmacodynamics” (14.89%, *n* = 7) and “Pharmacokinetics” (8.51%, *n* = 4) (Table 1, Fig. 16). This trend is also evident in the 100 most cited publications in *Naunyn–Schmiedeberg’s Archives of Pharmacology* from 1947 to 1974. The “Cholinergic and Adrenergic Systems” similarly dominated, comprising 21% of the research, followed by “Pharmacodynamics” and the “Dopaminergic System”—both contributing 14% (Basol and Seifert 2024). In contrast, among the Schmiedeberg Medal recipients, only one prize winner primarily focused on the “Dopaminergic System”, accounting for just 2.13%. This shows the significance of the research field “Cholinergic and Adrenergic Systems” also beyond the Schmiedeberg Medal recipients.

## Conclusions

As the most prestigious of all awards, the Nobel Prize attracts global attention, with its recognition extending far beyond any specific discipline. Numerous studies have been conducted on the winners of the Nobel Prize (Bünemann and Seifert 2024; Schlagberger et al. 2016). However, research on awards that are more specialized and limited to particular fields, like the Schmiedeberg Medal tend to receive less attention. While these prizes may not carry the same level of worldwide recognition, they are of significant value to the scientists who receive them. Statistically, the chance of winning a Nobel Prize is unrealistic for most researchers, whereas receiving a smaller, more focused award appears to be a far more attainable goal. Although these specialized awards may not enjoy the same global visibility, they play a crucial role within their respective fields. It is therefore important to explore these smaller awards, as they provide valuable insight into how research achievements are recognized at various levels.

To date, only one other study has focused on a specific DGPT award. Halling et al. analysed scientific prizes, paying particular attention to the gender distribution of the DGPT’s Fritz-Külz Prize, which we have discussed in greater detail in the “Gender” section (Halling et al. 2025).

While no additional studies on awards in German pharmacology have been conducted so far, comparisons can be drawn with the available award data in pathology. Malik et al. examined six pathology awards with a focus on gender. While the analysis found that, overall, the awards were distributed in accordance with the gender distribution among pathologists, it also revealed an underrepresentation of women in the allocation of more prestigious awards (Malik et al. 2024). This observation aligns with our own findings in the field of pharmacology.

Mispagel and Seifert also conducted an analysis of distinguished pharmacologists, focusing specifically on those who were persecuted in Germany between 1933 and 1945. They examined the careers of 71 pharmacologists, ten of whom were recipients of the Schmiedeberg Medal (Blaschko, Bülbring, Feldberg, Kosterlitz, Krayer, Loewi, Pick, Schild, Vogt, Wilbrandt). Their research revealed that the pharmacologists who emigrated during this period rarely published in *Naunyn–Schmiedeberg’s Archives of Pharmacology* after their emigration. Instead, their works were primarily published in the journals of their countries of exile. Our analysis similarly demonstrated a decline in the proportion of publication by prize winners in *Naunyn–Schmiedeberg’s Archives of Pharmacology*, highlighting the significant influence of World War II and the importance of Jewish scientists on the German pharmacological community (Mispagel and Seifert 2024).

To return to the original question regarding the circumstances that lead to the awarding of a prize, we can summarize: certain factors are not exclusive to Schmiedeberg Prize winners but are more prevalent among them, and a recipient of the Schmiedeberg Prize can typically be described as male, coming from an educated background, in good health, and enjoying a high standard of living. He has studied medicine, hails from Germany, and boasts a long and active career with high productivity in terms of publications. Nowadays, he publishes in English, and it is expected that he will also publish in *Naunyn–Schmiedeberg’s Archives of Pharmacology*. However, after the peak of his career, he will still have to wait some time until recognition in the form of the Schmiedeberg Medal.

We were also able to showcase the development of the scientific community over time: Researchers are increasingly working together in teams rather than individually, which has led to a significant rise in the number of publications. This reflects the scientific world that is both more fast-paced and increasingly international. In addition, English has emerged as the global language of science, further emphasizing the ongoing trend of internationalization.

## Limitations and future studies

We acknowledge the limitations of our study. Firstly, the analysis covers an extended period, during which many factors have influenced the careers of the prize winners to varying extents. These factors may have shaped each individual’s career trajectory in ways that are difficult to fully capture. Additionally, the prolonged observation period has affected both the availability and the quality of the data.

In addition, our study primarily examined a limited set of influencing factors. While these factors undoubtedly play a significant role in shaping the careers of the prize winners, there are numerous other variables that contribute to scientific success. These include factors such as mentorship, institutional support, access to research funding, and even the strategic decisions made by researchers throughout their careers. Moreover, external influences, such as political climates or religious ideologies, especially during turbulent periods like the World War II, have the potential to affect the direction of scientific careers. These elements remain underexplored in our study, and further research is needed to better understand their role in the long-term success of individual scientists.

Another key consideration is the rapid progress of digitalization in science. The technological landscape has evolved dramatically in recent years. These innovations have the potential to drastically reshape the pathways of future award winners, with emerging technologies providing both new opportunities and challenges. The increasing reliance on digital tools and collaborative networks across global platforms is likely to alter how research is conducted, published, and recognized. Understanding how these changes will impact the scientific careers of the future is an essential area of further investigation.

Looking ahead, future studies should consider the changing nature of scientific progress, the impact of digital transformation on research, and the influence of socio-political factors. This will enhance our understanding what drives scientific success and how prestigious awards, like the Schmiedeberg Medal, will evolve in an increasingly connected and rapidly changing world.

## Addendum: the Schmiedeberg Medal winners of 2025

Very shortly after completing this analysis, the Schmiedeberg Medal was awarded to Klaus Resch on March 26, 2025, at the 10 th German Pharm-Tox Summit in Hannover, Germany. Additionally, the award will go to Thomas Wieland in Mannheim, on April 9, 2025. To honour these two outstanding pharmacologists for their exceptional scientific contributions, we would like to provide a brief classification of their work in relation to our analysis. After acceptance of this paper, on May 8, 2025, the Schmiedeberg Medal was awarded to Urusla Ravens (Dresden). She is the 50th recipient of the Medal und the first woman since 1974 to receive this honour. Her scientific achievements will be summarized and honoured in a separate publication.

**Klaus Resch**

Klaus Resch was born in Berlin, Germany, in 1941. Coming from an academic background, he studied medicine in Freiburg, Germany, where he also began his career at the Max Planck Institute for Immunobiology. After completing his habilitation in 1974, he became an associate professor for Immunology at the University of Heidelberg. He was also a visiting scientist at the National Institutes of Health in the USA and at the Institute for Virology at the German Cancer Research Centre in Heidelberg. In 1981, he was appointed full professor of Molecular Pharmacology at the Hannover Medical School, the precursor to the Institute of Pharmacology, which he directed until his retirement in 2008. (Philippu 2014; Martin et al. 2025). Klaus Resch is currently 83 years old, which means he has far exceeded the life expectancy of his birth cohort (68.86 years). Throughout his scientific career, he published 264 papers with 467 co-authors, frequently as senior author (48.11%). The journal in which he published the most was the *Journal of Immunology* (9.09% of publications), reflecting his field of expertise. As an expert in immunopharmacology, he was a distinctive figure among pharmacologists. Notably, he never published in *Naunyn–Schmiedeberg’s Archives of Pharmacology*. This may be since during his active time as researcher, the journal published very little immunopharmacological research and had a strong focus on neurotransmitters. Based on the number of publications, the peak of his career occurred in 1989. Consequently, he had to wait for an impressive 36 years before receiving the Schmiedeberg Medal (Table S10).

**Thomas Wieland**

Thomas Wieland was born in 1960 in Karlsruhe, Germany, into a family of skilled occupation. He studied pharmacy in Heidelberg and later joined the Pharmacological Institute at the University of Heidelberg, where he earned his PhD and subsequently remained as a postdoctoral researcher. After positions at the University of Essen-Duisburg and the California Institute of Technology in Pasadena, USA, he was appointed Professor of Pharmacology at the University of Hamburg in 1997. In 2002, he returned to his Alma Mater at the Mannheim Medical Faculty (University of Heidelberg), where he currently holds the Chair of Experimental Pharmacology. He is also the Director of the Institute of Experimental and Clinical Pharmacology and Toxicology. His main research focuses on G-protein-mediated signal transduction in cardiovascular diseases (Philippu 2017; Lutz et al. 2025). He has made 261 publications so far, with an exceptionally high number of co-authors (*n* = 2291). He was the first author on 8.81% of these publications and the senior author on 19.92%. A notable portion of his work (8.05%) was published in *Naunyn–Schmiedeberg’s Archives of Pharmacology*, which leads the list of 111 journals in which his research has appeared. The peak of his career, based on his publication output, occurred in 2016. Following this peak, he had to wait 9 years before being honoured with the Schmiedeberg Medal, which he received at the age of 64 in Mannheim (Table S11).

In conclusion and based on our analysis, both Klaus Resch and Thomas Wieland fit well into the lineage of their highly renowned predecessors, each having made significant contributions to German and international pharmacology.

## Supplementary Information

Below is the link to the electronic supplementary material.

ESM 1 (DOCX 140 KB)

## Data Availability

All source data for this work (or generated in this study) are available upon reasonable request

## Abbreviations

DGPT: German Society for Experimental and Clinical Pharmacology and Toxicology (Deutsche Gesellschaft für experimentelle und klinische Pharmakologie und Toxikologie e.V.)
MHH: Hannover Medical School, Germany (Medizinische Hochschule Hannover)
n: Number
NSAP: Naunyn–Schmiedeberg’s Archives of Pharmacology
