# Correction: The Schmiedeberg Medal of the German Society for Experimental and Clinical Pharmacology and Toxicology: a biographical and bibliometric analysis of the 47 recipients from 1956 to 2024

**DOI:** 10.1007/s00210-026-05256-2

**Published:** 2026-03-24

**Authors:** Jessica Marie Steinert, Roland Seifert

**Affiliations:** Institute of Pharmacology, Hannover Medical School, Carl-Neuberg-Str. 1, D-30625 Hannover, Germany

**Correction: Naunyn-Schmiedeberg's Archives of Pharmacology (2025) 398:17239–17265**

10.1007/s00210-025-04260-2

In the original publication: *Steinert JM, Seifert R (2025) The Schmiedeberg Medal of the German Society for Experimental and Clinical Pharmacology and Toxicology: a biographical and bibliometric analysis of the 47 recipients from 1956 to 2024. Naunyn-Schmiedeberg’s Arch Pharmacol. *10.1007/s00210-025-04260-2*,* the birthplace of Franz Hofmann (Vienna) was incorrectly listed as Austria. At the time of his birth in 1942, Vienna was part of the German Reich (Germany). The corresponding passage should read as follows:

**Country of birth**

**Table 2:** Countries of birth of the prize winners: by number of prize winners

	Country of birth	Number of prize-winners	Percentage of prize-winners [%]
1	Germany	31	65.96
2	England	4	8.51
3	Austria	2	4.26
4	Sweden	2	4.26
5	USA	2	4.26
6	Czech Republic	1	2.13
7	Belgium	1	2.13
8	Netherlands	1	2.13
9	Ukraine	1	2.13
10	Greece	1	2.13
11	Croatia	1	2.13
	Total	47	100

The majority of prize winners (65.96%, 31 out of 47) were born in Germany, followed by prize winners from England (n = 4), Austria, Sweden, and the USA (n = 2) and Belgium, Greece, Croatia, the Netherlands, Czech Republic, and Ukraine (n = 1 each). This indicates that while the recipient of the Schmiedeberg Medal is expected to be a member of the DGPT, being born outside of Germany does not preclude one from receiving the award (Table 2).

This correction does not affect the main conclusions of the article. The authors apologize to Franz Hofmann and to the readers for this error.

