# Supplementary material for: The Schmiedeberg Medal of the German Society for Experimental and Clinical Pharmacology and Toxicology: a biographical and bibliometric analysis of the 47 recipients from 1956 to 2024
Source: Naunyn Schmiedebergs Arch Pharmacol. 2025 Jun 7;398(12):17239–65. doi: 10.1007/s00210-025-04260-2 (PMC12678592; doi:10.1007/s00210-025-04260-2)
Supplement: Supplementary file 1 — (DOCX 140 KB) [file 210_2025_4260_MOESM1_ESM.docx]

**The Schmiedeberg Medal of the German Society for Experimental and Clinical Pharmacology and Toxicology: a biographical and bibliometric analysis of the 47 recipients from 1956 to 2024**

**Jessica Marie Steinert, Roland Seifert**

Institute of Pharmacology

Hannover Medical School

Carl-Neuberg-Str. 1

D-30625 Hannover, Germany

Correspondence: [seifert.roland@mh-hannover.de](file:///C:\Users\Jessica\Downloads\seifert.roland@mh-hannover.de)

**Keywords**

*Schmiedeberg Medal, scientific award, prize winner analysis, bibliometric analysis*

**Supplemental figures and tables**

|  | Last name of prize winner | Sources of information |
| --- | --- | --- |
| 1 | Heubner | (Philippu 2017) |
| 2 | Loewi | [Curriculum Vitae Prof. Dr. Otto Loewi. Https://www.leopoldina.org/fileadmin/redaktion/Mitglieder/CV_Loewi_Otto_D.pdf . Accessed 28 Dec 2024 (Löffelholz and Trendelenburg 2008) (Philippu 2014)](http://www.leopoldina.org/fileadmin/redaktion/Mitglieder/CV_Loewi_Otto_D.pdf(Philippu%202014)(Löffelholz%20and%20Trendelenburg%202008)) |
| 3 | Pick | Ernst Peter Pick. In: Gegen das Vergessen. https://www.dgvs-gegen-das-vergessen.de/biografie/ernst-peter-pick/. Accessed 27 Dec 2024,  (Löffelholz and Trendelenburg 2008) |
| 4 | Dale | Archives of the DGPT: "Nachruf Sir Henry Dale" (ArchMHH Dep. 13 Nr. 31) Mitglieder. In: Nationale Akademie der Wissenschaften Leopoldina. https://www.leopoldina.org/mitgliederverzeichnis/mitglieder/member/Member/show/sir-henry-hallett-dale/. Accessed 28 Dec 2024 The Nobel Prize in Physiology or Medicine 1936. In: NobelPrize.org. https://www.nobelprize.org/prizes/medicine/1936/dale/biographical/. Accessed 28 Dec 2024 |
| 5 | Heymans | The Nobel Prize in Physiology or Medicine 1938. In: NobelPrize.org. https://www.nobelprize.org/prizes/medicine/1938/heymans/biographical/. Accessed 27 Dec 2024 |
| 6 | Liljestrand | Archives of the DGPT: "Lebenslauf Göran Liljestrand" (ArchMHH Dep. 13 Nr. 32) |
| 7 | Schmidt | Carl Frederic Schmidt. https://nap.nationalacademies.org/read/4990/chapter/14 . Accessed 28 Dec 2024 |
| 8 | Holtz | Archives of the DGPT: "Erinnerungen an Peter Holtz (1902-1970) in DGPT-Forum - Nr. 26 - Februar 2000" (ArchMHH Dep. 13 Nr. 30) Archives of the DGPT: "Lebenslauf Peter Holtz" (ArchMHH Dep. 13 Nr. 70) (Philippu 2017) |
| 9 | Krayer | (Löffelholz and Trendelenburg 2008) (Philippu 2021) |
| 10 | Schaumann | Archives of the DGPT: "Nachruf Otto Schaumann" (ArchMHH Dep. 13 Nr. 30) (Philippu 2021) |
| 11 | Verney | (Burgh Daly and Pickford 1997) |
| 12 | Burn | Archives of the DGPT: "Nachruf J.H. Burn" (ArchMHH Dep. 13 Nr. 31) (Bülbring and Walker 1984) (Rubin 2018) |
| 13 | v.Euler | Oswald Schmiedeberg Plakette verliehen. https://www.aerzteblatt.de/pdf.asp?id=210800 . Accessed 28 Dec 2024 The Nobel Prize in Physiology or Medicine 1970. In: NobelPrize.org. https://www.nobelprize.org/prizes/medicine/1970/euler/biographical/. Accessed 27 Dec 2024 |
| 14 | Feldberg | (Löffelholz and Trendelenburg 2008) (Philippu 2017)  Table S1: Sources of information regarding key data of the Schmiedeberg Medal prize winners in order of award |
| 15 | Brodie | Archives of the DGPT: "Nachruf Prof. Bernard Beryl Brodie Ph.D. in DGPT Mitteilungen - Nr. 6 - August 1990" (ArchMHH Dep. 13 Nr. 30) Bernard Beryl Brodie. https://prabook.com/web/bernard_beryl.brodie/123570. Accessed 27 Dec 2024 (Narvaez 1989) |
| 16 | Schulemann | Oswald Schmiedeberg Plakette verliehen. https://www.aerzteblatt.de/pdf.asp?id=210800 . Accessed 28 Dec 2024 (Philippu 2017) |
| 17 | Blaschko | (Born and Banks 1997) (Löffelholz and Trendelenburg 2008) (Rubin 2019) |
| 18 | Buelbring | (Löffelholz and Trendelenburg 2008) (Philippu 2017) |
| 19 | Dost | Archives of the DGPT: "Nachruf Friedrich Hertmut Dost in DGPT Forum - Nr. 26 - Februar 2000" (ArchMHH Dep. 13 Nr. 30) Friedrich Hartmut Dost. https://karger.com/che/article-pdf/22/2/73/2389264/000221914.pdf . Accessed Dec 28 2024 (Gladtke 1985) |
| 20 | Vogt | (Cuthbert 2005) (Löffelholz and Trendelenburg 2008) (Philippu 2017) |
| 21 | Kosterlitz | (Hughes 1996) (Löffelholz and Trendelenburg 2008) (North and Hughes 2013) |
| 22 | Wilbrandt | Archives of the DGPT: "Nachruf Walther Wilbrandt" (ArchMHH Dep. 13 Nr. 33) (Löffelholz and Trendelenburg 2008) |
| 23 | Schild | (Black 1997) (Löffelholz and Trendelenburg 2008) |
| 24 | Axelrod | (Snyder 2005) |
| 25 | Ariëns | Archives of the DGPT: "Curriculum virat E.J. Ariëns" (ArchMHH Dep. 13 Nr. 13/2) (Patil 2002) |
| 26 | Herken | Archives of the DGPT: "Lebenslauf H. Herken" (ArchMHH Dep. 13 Nr. 70) (von Bruchhausen 2003) |
| 27 | Kuschinsky | (Philippu 2017) |
| 28 | Remmer | Archives of DGPT: "Lebenslauf Herbert Remmer" (ArchMHH Dep. 13 Nr. 70) (Philippu 2017) |
| 29 | Fleckenstein | Archives of the DGPT: "Lebenslauf A. Fleckenstein, 24.12.1955" (ArchMHH Dep. 13 Nr. 70) (Mutschler and Friedrich)  Table S1(continued): Sources of information regarding key data of the Schmiedeberg Medal prize winners in order of award |
| 30 | Reuter | (Philippu 2014) |
| 31 | Hornykiewicz | (Philippu 2017) Archives of the DGPT: "Lebenslauf Oleh Hornykiewicz" (ArchMHH Dep. 13 Nr. 70) |
| 32 | Brock | Archives of the DGPT: "Prof. Dr. Dr. med h.c. Norbert Brock zum 70. Geburtstag" (ArchMHH Dep. 13 Nr. 13/1) Nachruf Prof. Dr. Dr. med. h.c. Norbert Brock \| BIOspektrum. https://www.biospektrum.de/magazinartikel/nachruf-prof-dr-dr-med-hc-norbert-brock. Accessed 28 Dec 2024 |
| 33 | Lembeck | (Philippu 2017) |
| 34 | Trendelenburg | Obituary Ullrich G. Trendelenburg, DrMed, Dphil. https://www.aspet.org/docs/default-source/news-files/the-pharmacologist/journal_search/v49n1_3_07.pdf?sfvrsn=72ff8bd2_0 . Accessed Dec 28 2024 (Philippu 2014) 1922 – 2006 Institut - Institut für Pharmakologie und Toxikologie. https://www.med.uni-wuerzburg.de/pharmatoxi/institut/. Accessed 27 Dec 2024 |
| 35 | Markwardt | (Philippu 2017) |
| 36 | Mutschler | Curriculum vitae Prof. Dr. rer. nat. Dr. med. Drs. h.c. Ernst Mutschler. https://historischesarchiv.dgk.org/files/2021/02/prof.-dr.-rer.-nat.-dr.-med.-drs.-h.c.-ernst-mutschler-2.pdf . Accessed Dec 28 2024 |
| 37 | Vater | (Beichler 2005) |
| 38 | Muschaweck | Ausstellungen. http://www.dphg2006.de/ausstellungen.htm. Accessed 27 Dec 2024,  Nachruf für Dr. med. Roman Muschaweck \| BIOspektrum. https://www.biospektrum.de/magazinartikel/nachruf-fuer-dr-med-roman-muschaweck?dl=1. Accessed Dec 28 2024 |
| 39 | Eichelbaum | Mitglieder. In: Nationale Akademie der Wissenschaften Leopoldina. https://www.leopoldina.org/mitgliederverzeichnis/mitglieder/member/Member/show/michel-eichelbaum/. Accessed 28 Dec 2024 (Philippu 2021) |
| 40 | Muscholl | Archives of the DGPT: "Lebenslauf Erich Muscholl (1964)" (ArchMHH Dep. 13 Nr. 70) Obituary Erich O.R. Muscholl. https://www.bps.ac.uk/getmedia/6bbabbd6-0081-4719-8290-a9bb649a59b2/Erich-Muscholl-obituary.pdf.aspx?ext=.pdf . Accessed Dec 28 2024 |
| 41 | Bock | Oswald-Schmiedeberg-Plakette 2012 für Karl Walter Bock. https://dgpt-online.eu/wp-content/uploads/2022/11/Zusammenfassung_BockKarlWalter.pdf . Accessed Dec 28 2024 (Philippu 2017) |
| 42 | Schroer | (Philippu 2014) |
| 43  Table S1(continued): Sources of information regarding key data of the Schmiedeberg Medal prize winners in order of award | Göthert | Mitglieder. In: Nationale Akademie der Wissenschaften Leopoldina. https://www.leopoldina.org/mitgliederverzeichnis/mitglieder/member/Member/show/manfred-goethert/. Accessed 28 Dec 2024 (Philippu 2014) |
| 44  Table S1(continued): Sources of information regarding key data of the Schmiedeberg Medal prize-winners in order of award | Schultz | Manfred Lindinger, Verflochtene Signalwege: Zum Tod des Biochemikers Günter Schultz, In: Frankfurter Allgemeine Zeitung vom 20. August 2021 Günter Schultz *23. Januar 1936 † 14. August 2021. https://www.fu-berlin.de/informationen-fuer/beschaeftigte/personalia/wir-trauern/media/Nachruf-Guenter-Schultz.pdf . Accessed Dec 28 2024 |
| 45 | Philippu | (Philippu 2014)  (2023) Schmiedeberg-Plakette für Athineos Philippou. https://www.uibk.ac.at/de/newsroom/2023/schmiedeberg-plakette-fur-athineos-philippou/. Accessed 28 Dec 2024 |
| 46 | Starke | Die DGPT gratuliert Herrn Prof. Dr. med. Klaus Starke; Freiburg, zum 80. Geburtstag \| BIOspektrum. https://www.biospektrum.de/magazinartikel/die-dgpt-gratuliert-herrn-prof-dr-med-klaus-starke-freiburg-zum-80-geburtstag. Accessed 28 Dec 2024 (Hein et al. 2024) Trauer um Prof. Dr. Klaus Starke — Medizinische Fakultät. https://www.med.uni-freiburg.de/de/fakultaet/aktuelles/Trauer_Starke. Accessed 28 Dec 2024 |
| 47 | Hofmann  Table S1(continued): Sources of information regarding key data of the Schmiedeberg Medal prize winners in order of award | Franz Hofmann. https://www.emeriti-of-excellence.tum.de/eoe/a-z/franz-hofmann/. Accessed 28 Dec 2024 Mitglieder. In: Nationale Akademie der Wissenschaften Leopoldina. https://www.leopoldina.org/mitgliederverzeichnis/mitglieder/member/Member/show/franz-hofmann/. Accessed 28 Dec 2024 |
|  |  |  |

| Predictor of latency | | | |
| --- | --- | --- | --- |
| Variable | Unstandardized | Standardized | Standard Error |
| (Constant) | 3,685 |  |  |
| Year of award | 0.009 | 0.014 | 0.09 |
|  |  |  |  |
| R Square | *0.000 |  |  |
| Adj. R Square | -0.022 |  |  |
| F (df=1; 45) | 0.009 |  |  |
| *p<0.05; **p<0.01; ***p<0.001;  Table S2: ANOVA (analysis of variance) test table – correlation of the latency from the peak of career to award and the year of award of the Schmiedeberg Medal | | | |

Figure S1: Scatter diagram of the latency from career peak to award in years by year of award

| Predictor of number of publications | | | |
| --- | --- | --- | --- |
| Variable | Unstandardized | Standardized | Standard Error |
| (Constant) | -7190.537*** |  |  |
| Year of award | 3726*** | 0.528*** | 0.893 |
|  |  |  |  |
| R Square | 0.279 |  |  |
| Adj. R Square | 0.263 |  |  |
| F (df=1; 45) | 17.417*** |  |  |
| *p<0.05; **p<0.01; ***p<0.001  Table S3: ANOVA (analysis of variance) test table – correlation of the number of publications and the year of award of the Schmiedeberg Medal | | | |

Figure S2: Scatter diagram of the number of publications by the prize winners by year of award

| Predictor of productivity (publication/year) | | | |
| --- | --- | --- | --- |
| Variable | Unstandardized | Standardized | Standard Error |
| (Constant) | *-131.094*** |  |  |
| Year of award | 0.068*** | 0.532*** | 0.016 |
|  |  |  |  |
| R Square | 0.283*** |  |  |
| Adj. R Square | 0.267*** |  |  |
| F (df=1; 45) | 17.746*** |  |  |
| *p<0.05; **p<0.01; ***p<0.001; | |  |  |

Table S4: ANOVA (analysis of variance) test table – correlation of the productivity (publications per year of active career) and the year of award of the Schmiedeberg Medal

Figure S3: Scatter diagram of the productivity (publication per year of active career) of the prize winners by year of award

| Document type | Number of publications | Percentage of publications [%] |
| --- | --- | --- |
| Article | 8256 | 88.78 |
| Review | 376 | 4.04 |
| Conference paper | 214 | *2.30 |
| Letter | 177 | *1.90 |
| Editorial | 72 | 0.77 |
| Note | 61 | 0.66 |
| Book chapter | 42 | 0.45 |
| Erratum | 40 | 0.43 |
| Short survey | 34 | 0.37 |
| Data not available | 23 | 0.25 |
| Book | 4 | 0.04 |
| Total | 9299 | 100 |

Table S5: Document types of the publications by the prize winners; by absolute number

| Predictor of number of co-authors | | | |
| --- | --- | --- | --- |
| Variable | Unstandardized | Standardized | Standard Error |
| (Constant) | -13592.721*** |  |  |
| Year of award | 6.941*** | 0.543*** | 1,601 |
|  |  |  |  |
| R Square | 0.295 |  |  |
| Adj. R Square | 0.279 |  |  |
| F (df=1; 45) | 18,8*** |  |  |
| *p<0.05; **p<0.01; ***p<0.001  Table S6: ANOVA (analysis of variance) test table – correlation of number of co-authors per prize winner and the year of award of the Schmiedeberg Medal | | | |

Figure S4: Scatter diagram of the number of co-authors of each prize winner by year of award of the Schmiedeberg Medal

| Coauthors of joint publications | Number of publications together |
| --- | --- |
| AXELRODJ. & BRODIEB.B. | 25 |
| BURNJ.H. & BÜLBRINGE. | 24 |
| KUSCHINSKYG. & MUSCHOLLE. | 14 |
| LILJESTRANDG. & V.EULERU.S. | 14 |
| KUSCHINSKYG. & MUTSCHLERE. | 13 |
| HOFMANNF. & SCHULTZG. | 11 |
| KUSCHINSKYG. & REUTERH. | 9 |
| LEMBECKF. & STARKEK. | 8 |
| BROCKN. & HERKENH. | 7 |
| DALEH.H. & FELDBERGW. | 5 |
| VERNEYE.B. & VOGTM. | 5 |
| BLASCHKOH. & BURNJ.H. | 4 |
| BOCKK.W. & EICHELBAUMM. | 3 |
| KOSTERLITZH.W. & KRAYERO. | 3 |
| BOCKK.W. & REMMERH. | 3 |
| FELDBERGW. & VOGTM. | 3 |
| MUSCHOLLE. & VOGTM. | 3 |
| DALEH.H. & FELDBERGW. & VOGTM. | 2 |
| FLECKENSTEINA. & MUSCHAWECKR. | 2 |
| ARIENSE.J. & MUTSCHLERE. | 2 |
| HERKENH. & REMMERH. | 2 |
| GÖTHERTM. & STARKEK. | 2 |
| BURNJ.H. & TRENDELENBURGU. | 2 |
| STARKEK. & TRENDELENBURGU. | 2 |
| KRAYERO. & VERNEYE.B. | 2 |
| DALEH.H. & VOGTM. | 2 |
| BLASCHKOH. & BÜLBRINGE. | 1 |
| BURNJ.H. & DALEH.H. | 1 |
| FELDBERGW. & KRAYERO. | 1 |
| HEYMANSC. & LILJESTRANDG. | 1 |
| MUSCHAWECKR. & MUTSCHLERE. | 1 |
| LOEWIO. & PICKE.P. | 1 |
| MUSCHOLLE. & SCHULTZG. | 1 |
| BLASCHKOH. & TRENDELENBURGU. | 1 |
| KUSCHINSKYG. & TRENDELENBURGU. | 1 |

Table S7: Number of publications in co-authorship of at least two Schmiedeberg Medal recipients, by absolute number of joint publications

| Year | Number of publications in NSAP | Number of publications in other journals | Total  number | Percentage of publications in NSAP  [%] |
| --- | --- | --- | --- | --- |
| 1893 | 1 | 0 | 1 | 100 |
| 1896 | 1 | 0 | 1 | 100 |
| 1898 | 0 | 2 | 2 | 0 |
| 1899 | 0 | 2 | 2 | 0 |
| 1900 | 1 | 2 | 3 | 33.33 |
| 1901 | 4 | 4 | 8 | 50 |
| 1902 | 2 | 0 | 2 | 100 |
| 1903 | 2 | 0 | 2 | 100 |
| 1904 | 0 | 2 | 2 | 0 |
| 1905 | 10 | 2 | 12 | 83.33 |
| 1906 | 0 | 2 | 2 | 0 |
| 1907 | 2 | 1 | 3 | 66.67 |
| 1908 | 6 | 0 | 6 | 100 |
| 1909 | 1 | 5 | 6 | 16.67 |
| 1910 | 3 | 6 | 9 | 33.33 |
| 1911 | 1 | 5 | 6 | 16.67 |
| 1912 | 5 | 5 | 10 | 50 |
| 1913 | 11 | 8 | 19 | 57.89 |
| 1914 | 3 | 5 | 8 | 37.5 |
| 1915 | 0 | 4 | 4 | 0 |
| 1916 | 2 | 3 | 5 | 40 |
| 1917 | 2 | 5 | 7 | 28.57 |
| 1918 | 5 | 2 | 7 | 71.43 |
| 1919 | 1 | 7 | 8 | 12.5 |
| 1920 | 2 | 15 | 17 | 11.76 |
| 1921 | 0 | 12 | 12 | 0 |
| 1922 | 1 | 25 | 26 | 3.85 |
| 1923 | 10 | 26 | 36 | 27.78 |
| 1924 | 3 | 36 | 39 | 7.69 |
| 1925 | 5 | 22 | 27 | 18.52 |
| 1926 | 3 | 27 | 30 | 10 |
| 1927 | 6 | 25 | 31 | 19.35 |
| 1928 | 7 | 22 | 29 | 24.14 |
| 1929 | 7 | 14 | 21 | 33.33 |
| 1930 | 13 | 14 | 27 | 48.15 |
| 1931 | 14 | 20 | 34 | 41.18 |
| 1932 | 5 | 25 | 30 | 16.67 |
| 1933 | 10 | 14 | 24 | 41.67 |
| 1934 | 3 | 26 | 29 | 10.34 |
| 1935 | 8 | 19 | 27 | 29.63 |
| 1936 | 15 | 23 | 38 | 39.47 |
| 1937 | 16 | 33 | 49 | 32.65 |
| 1938 | 12 | 27 | 39 | 30.77 |
| 1939 | 11 | 26 | 37 | 29.73 |
| 1940  Table S8: Number of publications by the prize winners in Naunyn-Schmiedeberg’s Archives of Pharmacology (NSAP) compared to the other journals combined, by year of publication | 1 | 31 | 32 | 3.13 |
| 1941 | 3 | 21 | 24 | 12.5 |
| 1942 | 3 | 33 | 36 | 8.33 |
| 1943 | 3 | 19 | 22 | 13.64 |
| 1944 | 1 | 17 | 18 | 5.56 |
| 1945 | 0 | 18 | 18 | 0 |
| 1946 | 0 | 43 | 43 | 0 |
| 1947 | 15 | 48 | 63 | 23.81 |
| 1948 | 5 | 60 | 65 | 7.69 |
| 1949 | 18 | 75 | 93 | 19.35 |
| 1950 | 28 | 88 | 116 | 24.14 |
| 1951 | 15 | 86 | 101 | 14.85 |
| 1952 | 19 | 104 | 123 | 15.45 |
| 1953 | 15 | 91 | 106 | 14.15 |
| 1954 | 21 | 123 | 144 | 14.58 |
| 1955 | 20 | 98 | 118 | 16.95 |
| 1956 | 25 | 90 | 115 | 21.74 |
| 1957 | 19 | 136 | 155 | 12.26 |
| 1958 | 20 | 133 | 153 | 13.07 |
| 1959 | 24 | 105 | 129 | 18.6 |
| 1960 | 25 | 120 | 145 | 17.24 |
| 1961 | 21 | 98 | 119 | 17.65 |
| 1962 | 29 | 118 | 147 | 19.73 |
| 1963 | 16 | 112 | 128 | 12.5 |
| 1964 | 32 | 98 | 130 | 24.62 |
| 1965 | 29 | 108 | 137 | 21.17 |
| 1966 | 51 | 115 | 166 | 30.72 |
| 1967 | 50 | 121 | 171 | 29.24 |
| 1968 | 42 | 125 | 167 | 25.15 |
| 1969 | 30 | 116 | 146 | 20.55 |
| 1970 | 21 | 124 | 145 | 14.48 |
| 1971 | 12 | 122 | 134 | 8.96 |
| 1972 | 10 | 113 | 123 | 8.13 |
| 1973 | 13 | 120 | 133 | 9.77 |
| 1974 | 45 | 142 | 187 | 24.06 |
| 1975 | 27 | 129 | 156 | 17.31 |
| 1976 | 21 | 103 | 124 | 16.94 |
| 1977 | 32 | 102 | 134 | 23.88 |
| 1978 | 27 | 99 | 126 | 21.43 |
| 1979 | 34 | 116 | 150 | 22.67 |
| 1980 | 40 | 134 | 174 | 22.99 |
| 1981 | 30 | 149 | 179 | 16.76 |
| 1982 | 18 | 143 | 161 | 11.18 |
| 1983 | 20 | 151 | 171 | 11.7 |
| 1984 | 21 | 129 | 150 | 14 |
| 1985 | 14 | 135 | 149 | 9.4 |
| 1986 | 18 | 120 | 138 | 13.04 |
| 1987 | 14 | 140 | 154 | 9.09 |
| 1988 | 23 | 153 | 176 | 13.07 |
| 1989  Table S8(continued): Number of publications by the prize winners in Naunyn-Schmiedeberg’s Archives of Pharmacology (NSAP) compared to the other journals combined, by year of publication | 26 | 126 | 152 | 17.11 |
| 1990 | 22 | 159 | 181 | 12.15 |
| 1991 | 19 | 165 | 184 | 10.33 |
| 1992 | 15 | 127 | 142 | 10.56 |
| 1993 | 16 | 101 | 117 | 13.68 |
| 1994 | 21 | 128 | 149 | 14.09 |
| 1995 | 24 | 120 | 144 | 16.67 |
| 1996 | 15 | 120 | 135 | 11.11 |
| 1997 | 18 | 93 | 111 | 16.22 |
| 1998 | 10 | 86 | 96 | 10.42 |
| 1999 | 21 | 103 | 124 | 16.94 |
| 2000 | 7 | 104 | 111 | 6.31 |
| 2001 | 7 | 81 | 88 | 7.95 |
| 2002 | 4 | 84 | 88 | 4.55 |
| 2003 | 2 | 101 | 103 | 1.94 |
| 2004 | 4 | 77 | 81 | 4.94 |
| 2005 | 2 | 76 | 78 | 2.56 |
| 2006 | 0 | 58 | 58 | 0 |
| 2007 | 1 | 51 | 52 | 1.92 |
| 2008 | 0 | 42 | 42 | 0 |
| 2009 | 1 | 50 | 51 | 1.96 |
| 2010 | 1 | 33 | 34 | 2.94 |
| 2011 | 0 | 42 | 42 | 0 |
| 2012 | 0 | 25 | 25 | 0 |
| 2013 | 0 | 33 | 33 | 0 |
| 2014 | 0 | 25 | 25 | 0 |
| 2015 | 0 | 27 | 27 | 0 |
| 2016 | 0 | 16 | 16 | 0 |
| 2017 | 0 | 22 | 22 | 0 |
| 2018 | 0 | 10 | 10 | 0 |
| 2019 | 1 | 9 | 10 | 10 |
| 2020 | 1 | 11 | 12 | 8.33 |
| 2021 | 0 | 11 | 11 | 0 |
| 2022 | 1 | 8 | 9 | 11.11 |
| 2023 | 3 | 2 | 5 | 60 |

Table S8(continued): Number of publications by the prize winners in Naunyn-Schmiedeberg’s Archives of Pharmacology (NSAP) compared to the other journals combined, by year of publication

|  | Language | Number of publications | % |
| --- | --- | --- | --- |
| 1 | English | 6721 | 72.28 |
| 2 | German | 2346 | 25.23 |
| 3 | undefined | 87 | 0.94 |
| 4 | Dutch | 61 | 0.66 |
| 5 | Polyglot | 29 | 0.31 |
| 6 | Italian | 17 | 0.18 |
| 7 | French | 12 | 0.13 |
| 8 | Swedish | 9 | 0.1 |
| 9 | Russian | 4 | 0.04 |
| 10 | Spanish | 4 | 0.04 |
| 11 | Japanese | 3 | 0.03 |
| 12 | Chinese | 2 | 0.02 |
| 13 | Czech | 1 | 0.01 |
| 14 | Danish | 1 | 0.01 |
| 15 | Norwegian | 1 | 0.01 |
| 16 | Polish | 1 | 0.01 |
|  | Total | 9299 | 100 |

Table S9: Distribution of languages used in the publications by the prize winners; by absolute numbers

Table S10: Summary of main bibliometric data of Klaus Resch (Source: www.scopus.com)

| Bibliometric data of Klaus Resch based on www.scopus.com | | |
| --- | --- | --- |
|  | Number of publications | Percent [%] |
| Total | 264 | 100 |
|  |  |  |
| Language of publication |  |  |
| English | 249 | 94.32 |
| German | 15 | 5.68 |
|  |  |  |
| Document type |  |  |
| Article | 247 | 93.56 |
| Conference paper | 7 | 2.65 |
| Review | 6 | 2.27 |
| Short survey | 2 | 0.76 |
| Erratum | 1 | 0.38 |
| Letter | 1 | 0.38 |
|  |  |  |
| Authorship |  |  |
| First author | 32 | 12.12 |
| Senior author | 127 | 48.11 |
|  |  |  |
| Five most frequent journals |  |  |
| Journal of Immunology | 24 | 9.09 |
| Journal of Biological Chemistry | 17 | 6.44 |
| European Journal of Immunology | 16 | 6.06 |
| BBA - Biomembranes | 13 | 4.92 |
| Immunobiology | 12 | 4.55 |

Table S11: Summary of main bibliometric data of Thomas Wieland (Source: www.scopus.com)

| Bibliometric data of Thomas Wieland based on www.scopus.com | | | | |
| --- | --- | --- | --- | --- |
|  | Number of publications | | | Percent [%] |
| Total | | 261 | 100 | |
|  | |  |  | |
| Language of publication | |  |  | |
| English | | 260 | 99.62 | |
| German | | 1 | 0.38 | |
|  | |  |  | |
| Document type | |  |  | |
| Article | | 226 | 86.59 | |
| Review | | 17 | 6.51 | |
| Book chapter | | 4 | 1.53 | |
| Editorial | | 3 | 1.15 | |
| Letter | | 3 | 1.15 | |
| Erratum | | 2 | 0.77 | |
| Short survey | | 2 | 0.77 | |
| Book | | 1 | 0.38 | |
| Conference paper | | 1 | 0.38 | |
| Data paper | | 1 | 0.38 | |
| Note | | 1 | 0.38 | |
|  | |  |  | |
| Authorship | |  |  | |
| First author | | 23 | 8.81 | |
| Senior author | | 52 | 19.92 | |
|  | |  |  | |
| Five most frequent journals | |  |  | |
| Naunyn-Schmiedeberg's Archives of Pharmacology | | 21 | 8.05 | |
| Journal of Biological Chemistry | | 15 | 5.75 | |
| American Journal of Human Genetics | | 13 | 4.98 | |
| Cellular Signalling | | 11 | 4.21 | |
| European Journal of Biochemistry | | 9 | 3.45 | |

**References**

Beichler C (2005) Das Leben und Wirken von Dr. Wulf Vater, 1st edn. Shaker, Aachen

Black JW (1997) Heinz Otto Schild, 18 May 1906 - 15 June 1984. Biographical Memoirs of Fellows of
 the Royal Society 39:381–415. <https://doi.org/10.1098/rsbm.1994.0022>

Born GVR, Banks P (1997) Hugh Blaschko, 4 January 1900 - 18 April 1993. Biographical Memoirs of
 Fellows of the Royal Society 42:40–60. <https://doi.org/10.1098/rsbm.1996.0004>

Bülbring E, Walker JM (1984) Joshua Harold Burn: 6 March 1892-13 July 1981. Biogr Mem Fellows R
 Soc 30:45–89. <https://doi.org/10.1098/rsbm.1984.0002>

Burgh Daly ID, Pickford LM (1997) Ernest Basil Verney, 1894-1967. Biographical Memoirs of Fellows
 of the Royal Society 16:523–542. <https://doi.org/10.1098/rsbm.1970.0022>

Cuthbert AW (2005) Marthe Louise Vogt. 8 September 1903 — 9 September 2003. Biographical
 Memoirs of Fellows of the Royal Society 51:409–423. <https://doi.org/10.1098/rsbm.2005.0027>

Gladtke E (1985) LaudatioProfessor Dr. Dr. Friedrich Hartmut Dost 75 Jahre. Klin Padiatr 197:375–
 375. <https://doi.org/10.1055/s-2008-1034007>

Hein L, Aktories K, Seifert R (2024) Obituary: Klaus Starke (1937–2024). Naunyn-Schmiedeberg’s
 Arch Pharmacol 397:2529–2530. <https://doi.org/10.1007/s00210-024-03040-8>

Hughes J (1996) Hans Kosterlitz (1903–96). Nature 384:418–418. <https://doi.org/10.1038/384418a0>

Mutschler E, Friedrich C (2020) Albrecht Vinzens Siegfried Fleckenstein (1917−1992) “Vater” der
 Calciumantagonisten. In Leuchttürme: Erfolgreiche Arzneimittelforscher im 20. Jahrhundert.
 Hirzel, Stuttgart

Narvaez AA (1989) Bernard B. Brodie, 81, a Pioneer In Drug Therapy Research, Dies. The New York
 Times

North RA, Hughes J (2013) Hans Walter Kosterlitz. 27 April 1903 — 26 October 1996. Biogr Mems
 Fell R Soc 59:171–192. <https://doi.org/10.1098/rsbm.2012.0037>

Patil PN (2002) Everhardus J. Ariëns (1918–2002): a tribute. Trends in Pharmacological Sciences
 23:344–345. <https://doi.org/10.1016/S0165-6147(02)02068->0

Philippu A (2021) Geschichte und Wirken der pharmakologischen, klinisch-pharmakologischen und
 toxikologischen Institute im deutschsprachigen Raum. Band 6: Autobiographien III und
 ausgewählte Biographien II. Berenkamp, Wattens

Rubin RP (2018) Joshua Harold Burn (1892–1981): A visionary during the evolution of pharmacology
 as a biomedical discipline. Journal of Medical Biography
 <https://doi.org/10.1177/0967772016685908>

Rubin RP (2019) Hermann (Hugh) Blaschko (1900-1993): His fundamental contributions to
 biochemical pharmacology and clinical medicine. J Med Biogr 27:179–183.
 <https://doi.org/10.1177/0967772017703091>

Snyder SH (2005) Julius Axelrod (1912–2004). Nature 433:593–593. <https://doi.org/10.1038/433593a>

von Bruchhausen: Nachruf Prof. Dr. med. Hans Herken. In: BIOspektrum. 9(3)/2003. Spektrum
 Akademischer Verlag, S. 291, ISSN 0947-0867
